# Long-Distance Dispersal Shaped Patterns of Human Genetic Diversity in Eurasia

**DOI:** 10.1093/molbev/msv332

**Published:** 2015-12-04

**Authors:** Isabel Alves, Miguel Arenas, Mathias Currat, Anna Sramkova Hanulova, Vitor C. Sousa, Nicolas Ray, Laurent Excoffier

**Affiliations:** ^1^Computational and Molecular Population Genetics Lab, Institute of Ecology and Evolution, University of Bern, Bern, Switzerland; ^2^Swiss Institute of Bioinformatics, Lausanne, Switzerland; ^3^Population and Conservation Genetics Group, Instituto Gulbenkian de Ciência, Oeiras, Portugal; ^4^Institute of Molecular Pathology and Immunology of the University of Porto (IPATIMUP), Porto, Portugal; ^5^Anthropology, Genetics and Peopling History Lab, Department of Genetics & Evolution-Anthropology Unit, University of Geneva, Geneva, Switzerland; ^6^EnviroSPACE Lab, Institute for Environmental Sciences, University of Geneva, Geneva, Switzerland

**Keywords:** human evolution, long-distance dispersal, last glacial maximum, out of Africa.

## Abstract

Most previous attempts at reconstructing the past history of human populations did not explicitly take geography into account or considered very simple scenarios of migration and ignored environmental information. However, it is likely that the last glacial maximum (LGM) affected the demography and the range of many species, including our own. Moreover, long-distance dispersal (LDD) may have been an important component of human migrations, allowing fast colonization of new territories and preserving high levels of genetic diversity. Here, we use a high-quality microsatellite data set genotyped in 22 populations to estimate the posterior probabilities of several scenarios for the settlement of the Old World by modern humans. We considered models ranging from a simple spatial expansion to others including LDD and a LGM-induced range contraction, as well as Neolithic demographic expansions. We find that scenarios with LDD are much better supported by data than models without LDD. Nevertheless, we show evidence that LDD events to empty habitats were strongly prevented during the settlement of Eurasia. This unexpected absence of LDD ahead of the colonization wave front could have been caused by an Allee effect, either due to intrinsic causes such as an inbreeding depression built during the expansion or due to extrinsic causes such as direct competition with archaic humans. Overall, our results suggest only a relatively limited effect of the LGM contraction on current patterns of human diversity. This is in clear contrast with the major role of LDD migrations, which have potentially contributed to the intermingled genetic structure of Eurasian populations.

## Introduction

Archaeological and genetic evidence suggest that anatomically modern humans (AMH) originated in Africa less than 200 Ka giving rise to all extant world populations through a major migration wave that left Africa around 60–70 Ka (Mellars 2006a, 2006b). Recently, whole-genome analyses of ancient DNA have also revealed that AMH admixed with archaic humans during their expansion out of Africa leaving a contribution of 1–6% in the genomes of extant Eurasians (Green et al. 2010; Reich et al. 2010, 2011). Although the evolutionary history of our own species is well known (Henn et al. 2012), many of its aspects like the number of colonization waves out of Africa, the exact migration routes followed by modern humans, or the effect of cultural developments on the genetic exchange and the inhabitable range of our species remain poorly understood. With the recent development of modeling techniques, the importance of the environment in shaping the distribution of human populations and, therefore, in conditioning the dispersal of our own species has been increasingly recognized (Ray et al. 2005; Banks et al. 2008; Eriksson et al. 2012; Stewart and Stringer 2012). For instance, climatic changes such as those driven by ice ages can deeply modify the spatial distribution and genetic diversity of a species (Hewitt 2000; Arenas et al. 2012). Even though AMH genetic diversity could have been impacted by cold periods (Hewitt 2000; Arenas et al. 2013), the specific genetic effect of the last glacial age that occurred between 13 and 29 Ka (Straus 1991) has been little investigated. Similar to what happened with many other species during the last glacial maximum (LGM) (Lahr and Foley 1998), modern humans might have retreated to refuge areas such as southern Europe as well as southern and south-eastern Asia (Banks et al. 2008; Stewart and Stringer 2012), and the extent of genetic differentiation between populations from different refugia might have increased during this period.

A consensual aspect of the migration process out of Africa is the relatively short time (∼10–15 Ky) it took for AMH to reach regions as remote as Southeast Asia and Oceania (see e.g., Mellars 2006a and references therein). It has been suggested that the early arrival of humans in Australia, which involved crossing water barriers, was only possible by the use of watercrafts such as boats or rafts (Stringer 2000; Balme 2013). Therefore, the migration of AMH to Oceania might have occurred through a form of long-distance dispersal (LDD). Archaeological evidence also suggests that artifacts associated with modern human behavior and hunting might have been spread over long distances, supporting likewise the existence of long-distance migration events during AMH history (McBrearty and Brooks 2000). Thus, although LDD has been associated to early modern behavior (Davidson and Noble 1992; Norton and Jin 2009), its role in the spread of Upper Paleolithic hunter-gatherer (HG) populations along the Eurasian (or American) coastlines is poorly understood. Similarly, its role in shaping human diversity has been little investigated as human colonization history has been mainly modeled under simple short-distance migration models (Hey 2005; Fagundes et al. 2007; Gutenkunst et al. 2009; Ray, Wegmann et al. 2010; Veeramah et al. 2012). Although a few studies have used spatially explicit models to describe the spread of humans (Prugnolle et al. 2005; Liu et al. 2006; Eriksson et al. 2012) and to take environmental heterogeneity into account (Ray et al. 2005), LDD events were not considered. In this study, we aim at better understanding which demographic forces have affected human evolution and to more precisely assessing whether LDD and climatic changes have played a significant role in shaping current patterns of human genetic diversity. We analyze a large population data set representative of Old World diversity under an approximate Bayesian computation (ABC) (Beaumont et al. 2002; Beaumont 2010) framework. This procedure allows us to estimate the relative probabilities of several scenarios of human dispersal out of Africa, ranging from a very simple spatial expansion to more complex models including LDD events and a LGM-induced range contraction (RC) or Neolithic-induced demographic expansions.

## Results

**Fig. 1.**
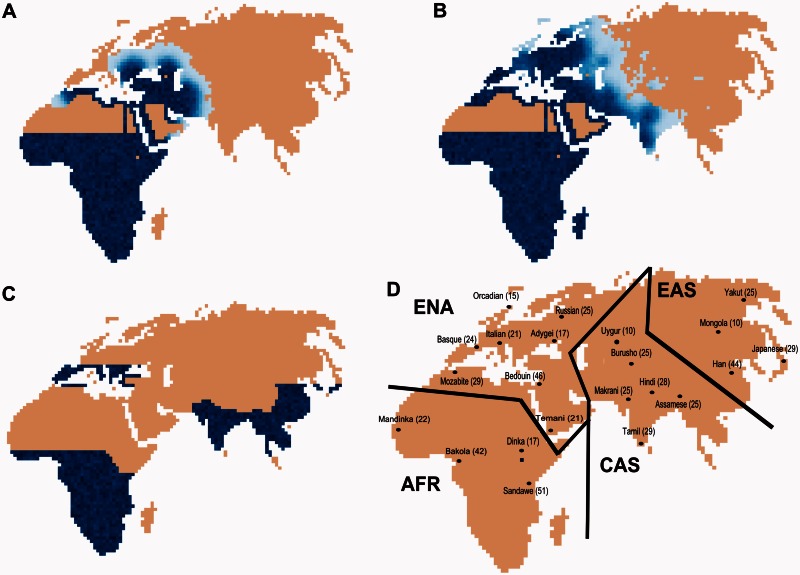
Main features of our spatially explicit simulations and sample locations. (*A*) Exit out of Africa through the Nile valley and along North African coastlines. (*B*) Colonization of Eurasia with visible LDD events ahead of the main wave front. (*C*) LGM refuge areas in Europe and Asia and contraction of sub-Saharan Africa due to the extension of the Sahara. (*D*) Locations and sizes of the 22 samples used in this study. The samples are found in four geographic regions abbreviated as AFR for sub-Saharan Africa, ENA for Europe, Near-East and North-Africa, CAS for Central Asia, and EAS for East Asia. The different shades of blue are proportional to population densities.

We integrated spatially explicit simulations into an ABC approach (Beaumont et al. 2002; Beaumont 2010) to assess whether LDD and LGM-induced RCs have shaped patterns of genetic diversity within and between human populations. Figure 1 shows key features of our tested spatially explicit scenarios, such as the initial Sub-Saharan range of modern humans (fig. 1A), the spread out of Africa into Eurasia with (fig. 1B) or without (fig. 1A) LDD events, and the potential Eurasian refuge areas during the LGM (fig. 1C).

We first tested four scenarios of modern human range expansion throughout the Old World. The simplest model (abbreviated as *noLDDnoRC*) consisted in a simple range expansion of modern humans from Eastern Africa (Prugnolle et al. 2005), where migrations only occurred between neighboring populations, and for which there was no LDD and no RC. The second model (abbreviated *noLDDRC*) is identical to the first one, except that we modeled an RC during the LGM toward southern refugia in Europe and Asia (as shown in fig. 1C). The third model (abbreviated *LDDnoRC*) is like the first model, except that LDD events occurred toward empty or already occupied demes. Finally, the fourth model (abbreviated *LDDRC*) is a model that included both LDD events and an RC (see Materials and Methods for more details on model implementation). Even though these four evolutionary scenarios are a great simplification of the real demographic history of our species, they should nevertheless capture key aspects of human evolution. To evaluate the relative fit of each of these four models, we looked at the molecular diversity at 87 microsatellite markers passing strict quality filtering (see Materials and Methods) across 22 populations selected to cover the Old World relatively evenly (as shown in fig. 1D). The relative posterior probability of each model was assessed under an ABC framework (Beaumont et al. 2002; Beaumont 2008). We thus fitted simulated microsatellite diversity at 50 loci with that observed at a thousand combinations of 50-loci resampled (bootstrapped) from the original set of 87 microsatellite markers (see Materials and Methods). Note that the computed model posterior probabilities are only relative to tested models and do not integrate over all possible models of evolution of AMH.

## Models without LDD Are Clearly Rejected

### Model Posterior Probability Distributions

**Fig. 2.**
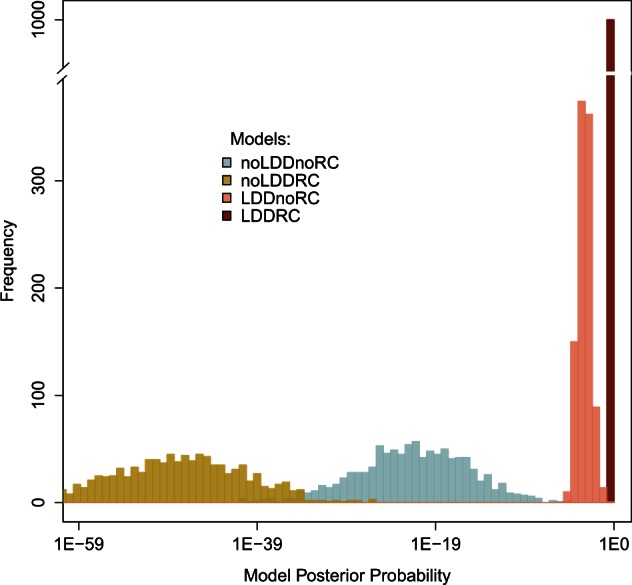
Distributions of the posterior probabilities of the four main scenarios of human expansions (*noLDDnoRC, noLDDRC, LDDnoRC,* and*LDDRC*) obtained over the 1,000 bootstrap data sets. Model posterior probabilities were computed using the multivariate logistic regression (Beaumont 2008) on the 2% best simulations (closest to the empirical data) among 100,000 simulations per evolutionary scenario.

Using a multivariate logistic regression approach (Beaumont 2008), we estimated the posterior probabilities of the four models described above for each of the 1,000 bootstrap data sets (see Materials and Methods). We find that the two evolutionary scenarios without LDD have extremely low posterior probabilities (median Pr(*noLDDnoRC*) = 3.38 × 10^−23^; median Pr(*noLDDRC*) = 1.74 × 10^−49^, fig. 2) and are thus clearly not supported by the data. Contrastingly, the *LDDRC* model, including both a LGM-induced RC and LDD, is very strongly supported by the data with posterior probabilities very close to one (median Pr(*LDDRC*) = 0.9995; bootstrap 95% CI: 0.9922–1.0000) and with the highest posterior probability among all 1,000 bootstrap data sets (fig. 2), whereas the *LDDnoRC* model, not including any LGM contraction, is much less supported (median Pr(*LDDnoRC*) = 0.0005, bootstrap 95% CI: 3.23 × 10^−05^–0.00789).

### Accuracy of Model Choice

We checked that our model choice procedure could correctly recover the true model by simulating genetic data sets (referred to hereafter as pseudo-observed data sets or PODS) under each model and estimating their posterior probabilities in the same way as for the observed data sets. We find that we can almost perfectly identify models without LDD (99.5% of correct identifications, supplementary table S1 and fig. S1, Supplementary Material online). For PODS generated under models with LDD, correct model identification drops to 86.8% and 87.9% for the *LDDnoRC* and *LDDRC* models, respectively (supplementary fig. S1 and table S1, Supplementary Material online), but in those two cases, the misidentified model is almost always (>99.5%) the alternative model with LDD. It implies that the presence or absence of the RC might be difficult to assess in presence of LDD. We also note that the proportion of correct assignment drops for higher levels of LDD (supplementary fig. S2, Supplementary Material online), suggesting that high frequencies of LDD can erase signals of RCs. The fact that data sets generated under models without LDD are never assigned to scenarios with LDD (supplementary table S1 and fig. S1, Supplementary Material online) suggests that LDD creates genetic signatures that cannot be reproduced by models without LDD, such as more uniformly distributed allele frequencies (see below). In addition, our model validation analysis reveals that when the threshold for model assignment is large (>85%) model miss-assignment is almost negligible (supplementary fig. S1 and table S1, Supplementary Material online), even between models including LDD.

### Goodness of Fit

**Fig. 3.**
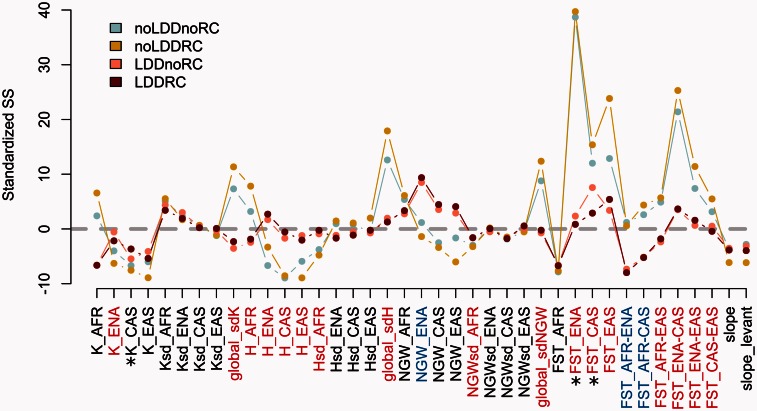
Fit between simulated and observed SSs for models *noLDDnoRC*, *noLDDRC, LDDnoRC,* and *LDDRC*. We report here, for each SS, the mode of the 2% simulations closest to the observations retained for posterior probability estimations. SSs were standardized using the mean and the standard deviation of each SS obtained across the 1,000 bootstrap observed data sets. Thus, SS modal values closer to zero (grey dashed line) indicate better fit to the observed SSs. The 39 SSs are fully described in supplementary table S4, Supplementary Material online. We have highlighted in red SSs where LDD scenarios were better supported by the data, and in blue SSs where no-LDD scenarios were better supported. SSs better supported by the *LDDRC* than by the *LDDnoRC* model are indicated by asterisks. All SS distributions are shown in supplementary figure S3*A*, Supplementary Material online.

We checked whether observed patterns of diversity could be reproduced under our simulated scenarios. We thus directly compared the observed summary statistics (SSs) to those generated under the different models. Overall, we find that most observed SSs can be reproduced under the four models (fig. 3, supplementary fig. S3*A*, Supplementary Material online). A closer examination of the SSs reveals that many of them are much better reproduced by models with LDD (SSs with red labels on fig. 3). Indeed, models with LDD allow populations to show higher levels of heterozygosity more similar to the observed values, as well as lower levels of population differentiation, as measured by *F_ST_,* within and between geographic regions outside Africa (fig. 3 red labels, supplementary fig. S3*B*, Supplementary Material online). On the other hand, models with LDD tend to erase signatures of bottlenecks as they cannot reproduce a statistic indicative of bottleneck (NGW, Garza and Williamson 2001) in Europe (NGW_ENA blue labeled on fig. 3), and they underestimate the amount of differentiation between Africa and Europe/Central Asia (FST_AFR-ENA and FST_AFR_CAS blue labeled on fig. 3, see also supplementary fig. S3*C*, Supplementary Material online). As shown in figure 3, adding a LGM-induced RC in the model with LDD (*LDDRC*) leads to a better fit of the average number of alleles in Central Asian populations (K_CAS), of FST among Central Asian populations (FST_CAS), and (to a lesser extent) of FST among European populations (FST_ENA). Indeed, European and Central Asian populations tend to be overly differentiated in models without RCs (see supplementary fig. S3*B*, Supplementary Material online). We also find that the inclusion of RCs in models with and without LDD tends to generate a steeper decrease in the average number of alleles per locus as one moves to higher latitudes, but this pattern does not allow us to discriminate between models (see supplementary fig. S4, Supplementary Material online). Finally, all models tend to generate a decay of population heterozygosity with distance from Africa, even though this decay is steeper than, but still overlapping with, the observed data (slope and slope_levant in supplementary fig. S3*A*, Supplementary Material online). In summary, the genetic signature of models with LDD are elevated levels of diversity within populations and low levels of population differentiation that are very consistent with patterns observed in the empirical data.

### LDD to Empty Habitats Was Disfavored during the Colonization of Eurasia

**Fig. 4.**
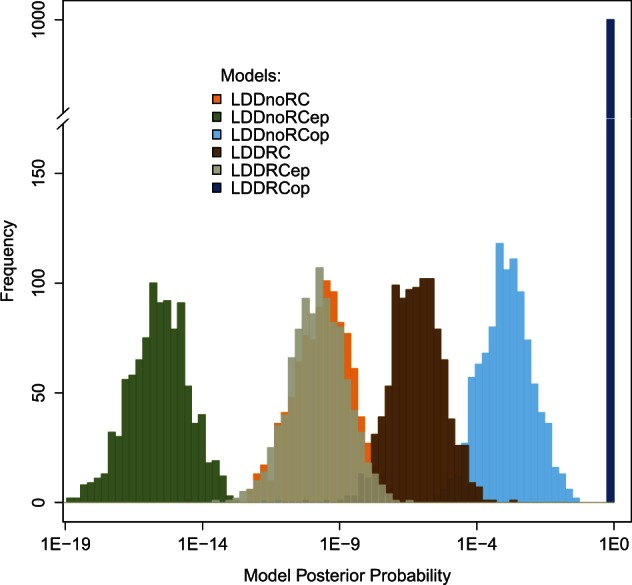
Distributions of the posterior probabilities of the six scenarios involving LDD (*LDDRC, LDDRCep, LDDRCop, LDDnoRC, LDDnoRCep,* and*LDDnoRCop*) obtained over the 1,000 bootstrap observed data sets. Model posterior probabilities were computed using the multivariate logistic regression (Beaumont 2008) on the 10% best simulations (closest to the empirical data) among 20,000 simulations per evolutionary scenario.

During a range expansion, LDD events can either lead to the colonization of a new subpopulation (deme) if migrants are sent to empty demes or lead to gene flow between already occupied demes. To assess which type of LDD events are important during the colonization phase, we contrasted the two previously favored *LDDRC* and *LDDnoRC* models with scenarios where LDD could only occur toward empty places (*ep) or only toward already occupied places (*op) (fig. 4) (see Materials and Methods for details). Interestingly, we find that models where LDD events are only occurring toward empty places are strongly disfavored and that the best supported model is one where LDD only occurs toward already occupied places (*LDDRCop*). This suggests that LDD events have been especially important to maintain genetic cohesion over long distances in already colonized regions of the world and that the colonization of Eurasia did not proceed by bursts of LDD. Moreover, the fact that a model with LDD events occurring only between occupied places is found vastly superior to *LDDRC* (where LDD can occur to both empty and occupied places) imply that LDD to empty habitats ahead of the wave front have been very rare during the colonization of Eurasia.

A model validation study shows that we have very high power (83.2%) to recognize the *LDDRCop* as the best model when it is true, and on average only ∼3.3% chance of misidentifying it as the best model when one of the other five models is true (see supplementary fig. S5 and table S2, Supplementary Material online). This result reinforces the view that LDD to empty places either did not occur at all or occurred at much lower rates than LDD between already occupied habitats. In addition, we find that our ability to distinguish this model from a similar one involving no LGM-induced RC is increased (supplementary fig. S5 and table S2, Supplementary Material online) as compared to the case where LDD occur to any deme. These results strengthen support for the existence of refuge areas into which many individuals migrated during the last LGM.

Although all six scenarios with LDD produced very similar levels of genetic diversity within populations (supplementary fig. S6*A*, Supplementary Material online), irrespective of how LDD was implemented, *op scenarios display better fitting values of *F_ST_* between non-African populations (supplementary fig. S6*B*, Supplementary Material online), and a more compatible extent of differentiation between African and European populations (F_ST__AFR-ENA, supplementary fig. S6*B*, Supplementary Material online). To access the goodness-of-fit of our LDD models and due to the high dimensionality of the SS space, we also examined the fit of the joint distribution of the simulated SSs to the empirical data by performing principal component analyses on the posterior distribution of the SSs generated under all models (“posterior predictive checks,” supplementary fig. S7, Supplementary Material online). Besides the general good fit of all scenarios involving LDD, on average the *LDDRCop* matches most of the six PCs combinations (2D *P* values ranging from 0.69 to 0.98) better than any of the remaining scenarios (*LDDnoRC* 2D *P* values: 0.65–0.88; *LDDnoRCep* 2D *P* values: 0.29–0.99; *LDDnoRCop* 2D *P* values: 0.54–0.98; *LDDRC* 2D *P* values: 0.53–0.85;*LDDRCep* 2D *P* values: 0.17–0.99, supplementary fig. S7, Supplementary Material online).

### Inferred Demographic Parameters

Most demographic parameters obtained under our best model (*LDDRCop,* table 1, supplementary fig. S8, Supplementary Material online) are in very good agreement with previous estimates obtained under different approaches and data sets. An ancestral size of about 12,000 individuals is virtually identical to that obtained by an ABC analysis (Wegmann et al. 2009) of tetranucleotide microsatellite diversity in Africa. A mean carrying capacity of about 1,036 is very close to a previously estimated average size for HGs of 839 (Hamilton et al. 2007) and within a previously estimated range of 600–1,200 (Deshpande et al. 2009). Moreover, assuming a 1:3 ratio for effective:census population size (Liu et al. 2006; Deshpande et al. 2009) and a deme size of 150 × 150 km, the reported *K* can be translated into a density of 0.14 individuals per km^2^, well within the 0.01–0.35 previously reported ranges for HG societies (Liu et al. 2006 and references therein). Our migration rate estimate of 0.15 is also close to the colonization rate of 0.13 estimated from the decay of heterozygosity with distance from Africa (Deshpande et al. 2009). Our estimate of migration rates, LDD proportions, and effective sizes suggest that about eight long-distance migrants (about 0.8% of the local effective size) left each deme per generation and travelled an average of 717 km. The trinucleotide mutation rate of 1.72e-4 is slightly lower than recent direct estimates obtained from pedigrees (2.7e-4 for dinucleotides, Sun et al. 2012) but in the range of previous estimates (0.9e-4–2.45e-4, Wegmann et al. 2009; Wegmann and Excoffier 2010). The time of exit out of sub-Saharan Africa (posterior mean = 66 Ka) is slightly older than previous estimates of 50–60 Ka, even though the lower limit of our confidence interval includes these more recent estimates (95% HPDI: 48–80 Ka). The estimated growth rate (*r*) of 0.56 per generation (95% HPDI: 0.20–0.92) is also in very good agreement to previous estimates (Eriksson et al. 2012) and, once more, well within the range estimated for HG populations (0.3 < *r*< 0.9, Liu et al. 2006 and references therein). Our estimate for the beginning of the colonization of Africa (mean = 95 Ka) by modern humans is more recent than those obtained by Eriksson et al. (2012) and considerably more recent than the oldest remaining of AMH found in Africa (Refs. 23 and 24 in Liu et al. 2006), nevertheless, the upper limit of our confidence intervals (95% HPDI: 80–121 Ka) overlaps with previous estimates obtained from genetic data (Voight et al. 2005; Fagundes et al. 2007).

**Table 1.** Demographic Parameters Estimated under the Best Fitting Model (*LDDRCop*).

Parameters	Mode	Mean	Median	95% HPDI^a^
Start of the initial expansion in Africa (*T_STARTEXP_*)^b^	80,704	94,903	91,777	80,000–120,916
Out of sub-Saharan Africa expansion time (*T_OOA_*)^b^	73,568	65,924	67,477	48,276–80,000
Ancestral size (Ne*_ANC_*)^c^	10,327	11,795	11,386	5,000–19,098
Carrying capacity (*K*)^c^	826	1,036	992	50–1,992
LDD proportion (LDD*_PROP_*)	0.044	0.038	0.040	0.021–0.050
Growth rate (*r*)	0.429	0.561	0.545	0.200–0.919
Average number of demes travelled by LDD migrants (µ)	5.357	4.780	4.946	3.074–6.000
Gamma shape parameter – LDD distance (α)	1.209	1.251	1.249	0.567–1.943
Migration rate (*m*)	0.110	0.155	0.148	0.050–0.268
Number of migrants (Nm)^c^	3	93	76	3–241
Number of LDD migrants (LDDNm)^c^	8	8	8	0–15
Mutation rate (STR_MUTRATE_)	1.74E-04	1.72E-04	1.72E-04	1.07E-04–2.36E-04

^a^HPDI, 95% highest posterior density interval.

^b^Times are in years, assuming a generation time of 25 years.

^c^Parameters estimates are reported in number of diploid individuals.

## Discussion

### Necessity and Costs of LDD during Human Evolution

LDD events have been often suspected in humans (Cavalli-Sforza et al. 1994) and even predicted from patterns of diversity (Lawson et al. 2012; Hellenthal et al. 2014). However, their importance in human evolution has never been formally tested in a spatially explicit context, and their global effect on patterns of human genetic diversity has not been examined in detail. We show here that models of human expansions in Eurasia without LDD events can be clearly rejected and that LDD events allowed human populations to maintain large levels of genetic diversity despite their relatively low densities before the Neolithic (table 1). Indeed, the occurrence of LDD migration appears to prevent large divergence between continental groups (supplementary fig. S3*B*, Supplementary Material online), and it might therefore partly explain why rare and young haplotypes are more often shared between rather than within populations, as recently reported (Mathieson and McVean 2014). Even though it is difficult to pinpoint what exactly triggered LDD events in prehistoric times (e.g., curiosity, inbreeding avoidance, changes in available resources, seasonality, and competition), it is worth noting that commercial trade could have promoted long-distance migrations, as attested by a recent study (Smith et al. 2015). Even though models including LDD are always more strongly supported than models without LDD, we find also strong evidence that no or very few migrants were sent ahead of the front during the colonization of Eurasia. Indeed, the best supported model is one where LDD would have only occurred between already colonized demes. This result might appear surprising, since higher dispersal abilities are supposed to evolve during range expansions (Phillips et al. 2010; Travis et al. 2010; Shine et al. 2011), but several factors could have prevented or severely limited these types of LDD. First, isolated long-distance migrants could have suffered from an Allee effect (Allee et al. 1949; Travis and Dytham 2002) (which is not implemented in our simulations) and be quickly exposed to strong inbreeding depression and a reduction in fitness, which would have prevented the emergence of fast growing colonies ahead of the main expanding wave front. On one hand, this is compatible with the results of a recent study showing that expanding populations show a decrease in fitness due to the accumulation of deleterious mutations on the edge of the expansion (Peischl et al. 2013). On the other hand, this is also in line with recent evidence showing that Allee effects can prevent loss of diversity in expanding populations (Roques et al. 2012), by allowing migrants from the core to restore diversity lost by gene surfing (Edmonds et al. 2004; Klopfstein et al. 2006). Second, long-distance migrants could have had problems in establishing successful colonies ahead of the modern human wave front if there had been strong competitions with archaic humans for accessing local resources. In this scenario, humans would have expanded only very progressively, and interactions with archaic humans would have been limited to the edge of the expanding front (Currat and Excoffier 2011). Note that a strong competition with archaic humans would only slightly slow down the human expansion wave (see table 1 in Currat et al. 2008), and low levels of archaic introgression, such as those found with Neanderthals in Eurasia (2–3%, Green et al. 2010; Reich et al. 2011; Prufer et al. 2014), would have very limited effects on the SSs used in this study. Overall, the fact that only certain types of LDD events might have been favored during (and after) human expansion suggests that LDD have evolved under opposite constraints. LDD within occupied areas would have restored diversity lost during range expansions and allowed populations to maintain sufficiently high local genetic diversity despite low effective size and an absence or very limited LDD events toward areas already occupied by other humans would have prevented direct competition with archaic populations. At this stage, it is still unclear if sporadic LDD events have occurred ahead of the expanding front but would have not left any trace in the current Eurasians.

This study has been performed by comparing the degree of genetic diversity in human populations genotyped for a relatively small number (87) of microsatellite markers. It would be interesting to reproduce our study and confirm our results by using genomic data sets, covering a much larger extent of human diversity. However, currently available large single-nucleotide polymorphism data sets (Li et al. 2008) suffer from unclear ascertainment bias that is impossible to reproduce in our simulations. Even though some efforts are put into the making of large collections of worldwide human DNA sequence, there is still no matching collection of unbiased genetic data that covers the Old World as evenly as this microsatellite database. Forthcoming whole-genome sequence data will certainly allow us to use linkage disequilibrium information, which has been shown to provide new insights on recent evolutionary events (Auton et al. 2009). However, linkage disequilibrium information was not used in this study since we analyzed a set of independent and unlinked loci. In any case, several studies have shown that microsatellites are informative for the demographic history of human (Ray et al. 2005; Ray, Wegmann et al. 2010; Eriksson et al. 2012) and nonhuman (Becquet and Przeworski 2007) populations. Moreover, our power studies show that these sets of 50 microsatellites can very well distinguish between models (supplementary figs. S1 and S5 and tables S1 and S2, Supplementary Material online), which makes us confident that our results would not drastically change if larger genomic data sets would be used in matching populations.

### A Recent Neolithic Population Size Increase Cannot Mimic the Effect of LDD Events

Since we have seen that the main effects of LDD events are to increase local diversity and reduce population divergence, one could wonder if a global recent increase in population size could not reproduce these patterns. Indeed, the increase in local population sizes that occurred after the Neolithic must have also increased the number of migrants exchanged between neighboring demes, which has been shown to increase local diversity and decrease *F_ST_* between populations (Excoffier 2004). To check if the implementation of a Neolithic population increase would make a model without LDD more compatible with the observations, we implemented a Neolithic-driven population growth in the *noLDDRC* model (see Materials and Methods for details) and estimated its posterior probability relative to the *noLDDRC* and the *LDDRC* models. Interestingly, we find that a model with a Neolithic expansion but without LDD has the lowest support (supplementary fig. S9, Supplementary Material online) and does not seem to improve the fit to the observed SSs (supplementary fig. S10, Supplementary Material online). This shows that our support for the occurrence of LDD events during the colonization of Eurasia is not due to the potential confounding effect of a widespread population growth. It also suggests that the loss of genetic diversity (and increased population differentiation) seen in models without LDD occurs very early during a range expansion and that a late Neolithic increase in population diversity cannot compensate this loss. We note that simulating a Neolithic expansion can sometimes improve the fit to the data but only when RCs and LDD events are also included in the model. However, this improvement is marginal and does not occur for all bootstrap data sets (data not shown)*.* It is also worth noting that we did not simulate here the effect of secondary range expansion of Neolithic farmers (NF) into HG territories. Previous simulations of this process have shown in the case of Europe that if NF and HG population had even small amount of interbreeding, the gene pool of the current population should be mainly of HG origin (Currat and Excoffier 2005; Currat et al. 2008). At odds with this finding, the first ancient DNA studies suggested that there had been an almost complete replacement of HGs by Near East farmers during the Neolithic in Europe (Bramanti et al. 2009; Malmstrom et al. 2009; Haak et al. 2010). However, this view has been revised as recent studies now show that there is a larger genetic dissimilarity between Early NF and modern Europeans than between Later NF and modern Europeans (Pinhasi et al. 2012; Bollongino et al. 2013; Lazaridis et al. 2014; Wilde et al. 2014; Haak et al. 2015). Also the Early NF influence is more visible in southern than in northern modern Europeans, which show stronger similarities with HGs (Skoglund et al. 2012; Haak et al. 2015). These observations are compatible with a scenario where 1) the northern Mediterranean coast acted as a migration corridor for NF (Pinhasi et al. 2012), and 2) Europe could have been recolonized from South to North establishing a gradient of genetic exchange between NF and HG populations (Skoglund et al. 2012). Although a massive migration of NF from the Middle-East and an almost complete replacement of HG populations would mimic the effect of LDD by reducing the overall genetic differentiation between European populations, current evidence suggests that this replacement did not really occur or that it was only a partial replacement with strong heterogeneities across Europe (Pinhasi et al. 2012; Skoglund et al. 2012; Lazaridis et al. 2014; Wilde et al. 2014; Haak et al. 2015). The settlement of Europe has thus been more complex than previously anticipated, with even some contribution of a remote Western Asian population into Europe (Haak et al. 2015), potentially by an LDD event. However, it would appear very difficult to accurately model all the region-specific details of the settlement of Europe, a task that would be even more challenging in other regions were the amount of information on past demographic events is even lower. Our spatially explicit simulations with LDD, which do not introduce a strong discontinuity between HG and NF populations, appear nevertheless as a reasonably good approximation of the evolutionary history of Eurasia.

Note that we have not only tested the effect of larger deme carrying capacities but also of larger deme physical dimensions on the model choice procedure, since LDD could be artificially favored by our model choice procedure if the dimension of the demes is too small in our simulations. To explicitly test this possibility, we performed simulations under the *noLDDRC* and *LDDRC* models with demes occupying areas of 250 × 250 km (instead of 150 × 150 km), and we adjusted their carrying capacity to keep the same density per square km. We then computed the posterior probabilities of these new models relative to those with the original deme dimensions. As expected, increasing deme dimensions increases the average posterior probability of the *noLDDRC* scenario (supplementary fig. S11, Supplementary Material online), but it also increases the posterior probability of the *LDDRC* scenario, which remains the overall best model. Although a larger deme dimension improves the posterior probability of all models, we note that a 250-km-wide deme size is much larger than any previous estimates of deme dimensions reported for HG populations (Hewlett et al. 1982; Ammerman and Cavalli-Sforza 1984; Gronenborn 1999; Anderson and Gillam 2000), and it is 2.5 times larger than deme dimensions used in previous spatially explicit simulations studies (Ray et al. 2005; Eriksson et al. 2012). Since scenarios with LDD are better supported even with large deme sizes, our conclusion that LDD events are necessary to explain patterns of human diversity should remain valid.

### Evidence for LGM-Induced RCs in Eurasia

Our results suggest that the LGM only marginally affected patterns of human genetic diversity. This is in line with the observation that the effect of RCs is only obvious when there is little gene flow between neighboring demes (Arenas et al. 2012), and subsequent LDD events could have thus lessen the effects of past RCs. Although our model choice procedure provides support for models involving both LDD events and RCs, RCs do not seem to have a very pronounced effect on the SSs when LDD events are implemented, with the exception of the number of alleles found in Central Asians, the average *F_ST_* between Central Asian populations and to a less extent the average *F_ST_* among Europeans populations (fig. 3, SSs indicated with asterisks). We also looked at the fit between simulated and observed data by considering pairs of SSs rather than considering single SSs, that is, we examined two-dimensional distributions of SSs. Using this approach, we find that among 364 2D SS distributions for which at least one model generates SS joint distributions similar to the empirical data, the *LDDRC* model performed better in 152 cases, as compared to 119 cases for the *LDDnoRC* model (see Materials and Methods for a detailed description of these computations). The *LDDRC* scenario thus generates 2D SS modal values that are more often closer to the centroid of distributions obtained by bootstrapping the observed data, but this better fit is relatively marginal, suggesting that the overall better fit of the scenario involving RCs emerges by considering all statistics collectively. We note, however, that, after restricting LDD events to occupied places, the relative support of scenarios with RCs increases (compare figs. 2 and 4) and the power to correctly identify such a scenario when it is true is still larger than 95% (using a 0.85 posterior probability threshold—supplementary table S2, Supplementary Material online). Simulated *F_ST_* values within Europe, within central Asia, and between Europe and Central Asia are also better fitting observations when RCs are implemented. Nevertheless, the fact that there is a larger difference between statistics generated with and without RCs in the absence of LDD (supplementary fig. S3*A* and *B*, Supplementary Material online) and that long-distance migrations decrease our power to detect RCs (supplementary fig. S2, Supplementary Material online), suggest that LDD events can partially erase the signature of previous RCs. We also note that our implementation of RC is very simple and quite arbitrary, as there is still a lot of uncertainties on the exact place of refuge areas during the LGM and on the dynamics and timing of these contractions. Along these lines, although the spatially explicit range expansion models tested here are more realistic than those implemented in previous studies (but see Eriksson et al. 2012, for another spatially explicit simulation framework), they are still very simplistic relative to the likely complex demographic history of humans (Henn et al. 2012). Additional sources of realism could include temporal and regional differences in local population sizes and migration rates related to environmental heterogeneity (Ray et al. 2008; Mona et al. 2014), biased sex dispersal (Benguigui and Arenas 2014), a decoupling of migration and colonization rates (Deshpande et al. 2009), or kin and mass migration (Fix 2004). However, such complex models could become rapidly over determined and more difficult to explore due to the large number of extra parameters to examine.

## Conclusions

Although we find that LDD ahead of the wave front were not very important during the settlement of Eurasia, this conclusion might not be valid for all continents. There is indeed some evidence that Oceania has been colonized >50 Ka by long-distance migration events, probably by people using boats to reach Australia (Stringer 2000; Balme 2013). Dispersal by boat has been used more recently for the colonization of the Pacific Ocean, and there are indications that the fast colonization of the Americas was made possible by fast coastal migrations (Wang et al. 2007; Reich et al. 2012), potentially over long distances. However, the fact that regions in which there is evidence for long-distance migrations during the colonization process were devoid of any archaic humans (Oceania, Polynesia, and the Americas) is in line with the view that LDD events ahead of the colonization front might have been prevented or limited by interspecific competition. It suggests that intrinsic effects such as inbreeding depression might not have been the main factors preventing LDD events ahead of the expansion front. Therefore, the inland colonization of Eurasia has been a quite progressive process, where serial founder effects (Henn et al. 2012) and gene surfing (Excoffier et al. 2009) might have led to a progressive loss of diversity with distance from Africa (Prugnolle et al. 2005). However, LDD events from the core to the front might have quickly restored diversity and reshuffled the genetic diversity of populations in Eurasia. These LDD events might also explain why the gene pool of many human populations shows signals currently interpreted as admixture events between isolated populations (Patterson et al. 2012; Moorjani et al. 2013; Pickrell et al. 2014) that could just represent normal patterns having been built since the exit of modern humans from Africa.

## Materials and Methods

### Population Samples and Genetic Marker Selection

Patterns of human genetic variation were summarized by selecting 22 worldwide populations from a larger microsatellite data set including 848 loci (Tishkoff et al. 2009). The 22 populations were selected to have at least 15 sampled individuals (except Mongola and Uygur, with *n* = 10) and to have a relatively even geographic distribution over the Old world (fig. 1D). We considered here only trinucleotide microsatellites to prevent a putative ascertainment bias effect recently reported for tetranucleotide repeat loci (Eriksson and Manica 2011). In addition, we filtered out those loci showing properties that did not fit the stepwise-mutation model used in our simulations (e.g., showing evidence of indels or incomplete repeated motifs). We also removed loci with >5% missing data as well as compound microsatellites (Pemberton et al. 2009). This resulting set of 87 microsatellites is referred to hereafter as the “filtered data set.”

### Demographic Simulations

An extended version of SPLATCHE2 (Ray, Currat et al. 2010) was used to simulate the colonization of AMH across the Old World, implementing LDD as well as RC (Ray and Excoffier 2010; Arenas et al. 2012). SPLATCHE is a two-step simulator, in which the first-step concerns the simulation (forward in time) of the population demography, that is, deme growth and migration history, and the second step consists in generating genetic diversity via coalescent simulations using the information recorded during the demographic step. Therefore, genetic drift occurs through the process of random sampling within the coalescent simulations (Excoffier et al. 2009; Ray, Currat et al. 2010). We generated land surface by using the Hammer-Aitoff projection, which divides the Old World into 9,047 squared demes (subpopulations) of 22,500 km^2^ each. Land bridges connecting the continent to some islands, such as Great Britain and Japan, have been artificially added to allow the settlement of these regions in scenarios without LDD.

We assumed that the range expansion started some *T_STARTEXP_* generations ago from a single deme (located in Addis Ababa, Ethiopia, following (Pritchard et al. 2000) assumed to have an ancestral population size equal to Ne*_ANC_* diploid individuals. Each generation, deme density is logistically regulated, with an intrinsic growth rate (*r)* and a carrying capacity (*K),* followed by a migration phase, in which 2*N_t_m* migrants are sent to the four neighboring demes, where *N_t_* stands for the haploid number of individuals within the deme at generation *t*. All model parameters are drawn from prior distributions shown in supplementary table S3, Supplementary Material online.

#### Long-Distance Dispersal

In our simulation framework, LDD events represent migration events to nonadjacent demes. The total number of long-distance (LD) migrant genes is drawn from a binomial distribution *b*(2*N_t,_ m,* LDD*_PROP_*), where LDD*_PROP_*is the proportion of the total number of migrant genes (2*N_t_m)* that are long-distance dispersers. Once the number of LD migrant genes is computed each LD migrant moves X demes away, where is sampled from a gamma distribution Gamma(α, α/$\overset{-}{\mu }$) where α is the shape parameter and $\overset{-}{\mu }$ is the average distance travelled by the migrant individuals. Both α and $\overset{-}{\mu }$ are hyperparameters drawn from uniform priors (see below), which ranges produces distributions ranging from exponential to almost normal. Therefore, our method is able to cover alternative LDD kernels. To avoid unrealistically long LD migration events, we arbitrarily set the maximum dispersal distance to 6 demes (900 km). See Ray and Excoffier (2010) for a more detailed description of the LDD distribution kernel.

### LGM Range Contraction

The RC underlying the LGM was modeled by shrinking progressively the range of habitable areas for human populations. To model a LGM-induced RC, the onset of the RC (*T_SCONTR_*) was set to 1,000 generations ago or 25,000 BP (assuming a 25-year generation time), and its dynamics was modeled by simulating 30 RC events, two or four generations apart, in which two or one latitudinal layer of demes become uninhabitable by setting their carrying capacity to zero (see Arenas et al. 2012 for more details on this process). The number of generations between each contraction event and the width of each contraction (in number of demes) were chosen to fit the potential duration of the contraction process (120 generations or 3,000 years) and the potential geographical location of the refugia (see below). Populations were then restricted to refugia shown in figure 1C for 160 generations (4,000 years) and then free to migrate again to recolonize the Eurasian landmass. The refuge areas suitable for humans during the LGM were derived from paleo-vegetation maps of the LGM (Ray and Adams 2001). They consisted in 1) Southern Europe, represented as a continuum from the Iberian Peninsula to the Turkish coast; 2) a very narrow strip over the northern most part of Africa; 3) India and South Eastern China, which were also modeled as a continuous region; and 4) sub-Saharan Africa.

### Coalescent Simulations

During the forward demographic simulations, we recorded at each generation the deme densities, as well as the number of LDD and non-LDD immigrants. This demographic database was then used to perform coalescent simulations backward in time and to generate genetic data. Because of the computational cost of our spatially explicit simulations, we simulated multilocus genotypes for only 50 microsatellite loci, matching the number of individuals per sampled population (fig. 1D), assuming a strict stepwise mutation model and a mutation rate at all loci drawn from a uniform prior STR_MUTRATE_ ∼ U[5 × 10^−5^–3 × 10^−4^] (supplementary table S3, Supplementary Material online).

### Main Tested Evolutionary Scenarios

We first examined four alternative scenarios including LDD and RC or not. The simplest model examined in this study involves a single range expansion with no LDD and no RC (hereafter referred to as *noLDDnoRC*). It posits that modern humans emerged in Eastern Africa 3,200–6,000 generations BP (80–160 Ka). They then spread across Sub-Saharan Africa, where they remained until 1,200–3,000 generations (30–75 Ky) before being allowed to migrate along the Nile river valley and the Red Sea coast to colonize the Eurasian continent (the Out-of-Africa event). Then, individuals could migrate over the Old World except the Arabic desert and the Himalayas, where carrying capacities were set to zero. We implemented two additional models derived from the *noLDDnoRC* model, by adding either a LGM-induced RC (the *noLDDRC* model) or LDD events (the *LDDnoRC* model), as described above. The fourth tested model includes both a RC and LDD events and is abbreviated as*LDDRC.* Note that here LDD migrants can be sent either to an empty or to an occupied deme, the former case being a founding event. All demographic parameters such as carrying capacities (*K*), growth rate (*r*), migration rate (*m*), LDD proportion (LDD*_PROP_*), and all the parameters related to the movement of LDD migrants are drawn from prior distributions shown in supplementary table S3, Supplementary Material online. Note that all parameters related to the RC were fixed.

### Additionally Tested Scenarios

To see whether the Neolithic and its associated population size increase could generate patterns similar to those produced by LDD, we extended the *noLDDRC* to include a sudden population size increase 320 generations (8,000 years) ago. We thus included an additional parameter (*KNeo*∼U [2,500–7,500], supplementary table S3, Supplementary Material online) representing the carrying capacity of post-Neolithic populations.

We also studied two alternative scenarios with LDD, where 1) LDD could only occur toward empty places (demes), implying they would always correspond to a colonization event and 2) LDD could only occur toward already colonized demes. We use the *ep (empty places) and *op (occupied places) suffixes to refer to these two scenarios.

### Approximate Bayesian Computations

We used ABC (Beaumont et al. 2002; Beaumont 2010) to compute the relative posterior probabilities of the tested models via simulations. Genetic data sets matching the sample composition and the number loci used in this study were generated by 1) drawing parameter values from prior distributions (supplementary table S3, Supplementary Material online); 2) performing demographic and coalescent simulations using SPLATCHE 2 (Ray, Currat et al. 2010); and 3) recording SSs computed on simulated genetic data using arlsumstat (Excoffier and Lischer 2010). All these steps were performed 100,000 times for each of the four main models using ABCtoolbox (Wegmann et al. 2010). Because of the large computational costs of our simulations, other model comparisons were performed using fewer simulations. SSs and model choice procedure are explained below.

### Summary Statistics

Simulated and observed genotype data were summarized by computing for each population the mean and the standard deviation (SD) over loci of the following statistics: number of alleles (*K*), heterozygosity (*H*) and modified Garza-Williamson statistics *NGW*) (Garza and Williamson 2001). The SD of these SSs was also computed after pooling all samples (supplementary table S4, Supplementary Material online). Additionally, we computed *F_ST_* between all pairs of populations taking into account the difference in number of repeats between microsatellite alleles (*R_ST_*). To reduce the number of SS, we averaged the SSs within four geographically defined groups of populations (fig. 1D, main text). Pairwise *R_ST_* were also averaged within and between population groups. We measured the decay of population heterozygosity with least-cost geographic distance from the origin of the expansion by computing the slope of the fitted linear regression. Since with LDD, migrant genes can cross short sea tracts (e.g., migrate directly from the Horn of Africa to the Arabian Peninsula, or from Morocco into the Iberian Peninsula), we computed the slope of the regression on two geographic distance matrices. In the first matrix, geographic distances between populations were computed assuming that migrants could only migrate out of Africa through the Levant, and in the second matrix, distances were computed by allowing migration out of Africa also through the Horn of Africa and Gibraltar. The 39 SSs used to perform ABC model choice are listed in supplementary table S4, Supplementary Material online.

#### Model Choice

To perform model choice, we first computed the Euclidian distance between the observed and simulated SS under the different models and then retained the 2–10% (tolerance) of the simulations closest to the observed data set, depending on the total amount of simulations performed under different models. We then computed model posterior probabilities using the multivariate-weighted logistic regression (MLR), introduced by Beaumont (Fagundes et al. 2007; Beaumont 2008), where the model (*M*) indicator (1,.,*n_M_*) is considered a categorical variable Y and logistic regression coefficients ${(\beta }_{j}^{T})$ for the model *j* are estimated in $P(Y=j|\;S)=\frac{e^{{(\beta }_{j}^{T}S)}}{\sum_{i=1}^{M}e^{\left(\beta_{i}^{T}S\right)}}$, where *S* are the observed SSs. The functions vglm() and predictvgml() from the R package VGAM were used to compute the fitted coefficients and model posterior probabilities, respectively (see the following references Fagundes et al. 2007; Beaumont 2008 for more details). The MLR was performed on 1,000 observed data sets obtained by resampling 50 microsatellites without replacement from the “filtered data set,” providing confidence intervals for these posterior probabilities representing the influence of the choice of 50 microsatellites on our results. The MLR was computed with the R function *Calmod*, developed by M. Beaumont and available on https://code.google.com/p/popabc/ (last accessed December 15, 2015).

#### Parameter Inference

Parameters of the *LDDRCop* model were estimated via a conventional ABC procedure (Leuenberger and Wegmann 2010; Wegmann et al. 2010). Partial least square (PLS) components were first computed on Box-Cox transformed SSs to decrease the dimensionality of the SS space and to maximize the linearity between SS and parameters. The retained number of PLS components (12) was chosen such that the addition of more PLS would not decrease the root mean squared error (RMSE, see Ray, Wegmann et al. 2010 for more details and supplementary fig. S8, Supplementary Material online). Parameter posterior distributions were computed from the 2,000 simulations (out of 50,000 simulations) closest to the observed data and under a general linear model approximating the likelihood function, as implemented in ABCtoolbox (Wegmann et al. 2010).

#### Power of the Model Choice Procedure

To test if we have enough power to distinguish between alternative models, we used 1,000 randomly chosen simulations, referred to hereafter as PODS, and computed model posterior probabilities for each PODS, as described above. A PODS was assigned to one of the models if its relative probability was larger than either 50% or 85%, and considered unassigned otherwise. This model validation procedure was carried out for the four main alternative models (supplementary fig. S1, Supplementary Material online) and for the six scenarios with different types of LDD events (supplementary fig. S5, Supplementary Material online).

#### Goodness of Fit

We investigated the fit between the empirical (bootstrap replicates) and the simulated SSs generated under all models tested in this study. The fit was investigated by looking at individual SSs (fig*.* 3, supplementary figs. S3*A*–*C*, S6*A* and *B*, S10, and S12, Supplementary Material online) and/or at the 2D joint distributions of all pairwise combinations of the different SSs (data not shown). When investigating 2D joint distributions, we computed the 2D densities across the 1,000 observed bootstrap replicates and then computed the *P* values of the 2D highest density points (mode) obtained from the 2D joint distribution of the SSs recovered by retaining the best 2% simulation generated under alternative models. The 2D densities and *P* values were computed following Daub et al. (2014). R scripts to perform these analyses are available upon request.

We also performed posterior predictive checks to verify that the selected scenarios can reproduce the empirical data. We first performed parameter inference using the same procedure as for the *LDDRCop* model, described above. We then sampled 1,000 parameter vectors from the obtained posterior distributions and used them as input parameters for SPLATCHE2 (Ray, Currat et al. 2010) to generate 1,000 new genetic data sets for each demographic scenario. These “posterior data sets” were then summarized via principal component analysis in the case of the six models with LDD. The first four PCs were plotted against each other, and for each 2D plot, we projected the observed data set and computed the curves for the 95%, 75%, and 50% envelopes of the posterior 2D predictive distributions (supplementary fig. S7, Supplementary Material online). The 2D densities and *P* values were computed as described above.

## Supplementary Material

Supplementary figures S1–S12 and tables S1–S4 are available at *Molecular Biology and Evolution* online (http://www.mbe.oxfordjournals.org/).

Supplementary Data
