# Supplementary material for: Long-Distance Dispersal Shaped Patterns of Human Genetic Diversity in Eurasia
Source: Mol Biol Evol. 2015 Dec 4;33(4):946–58. doi: 10.1093/molbev/msv332 (PMC4776706; doi:10.1093/molbev/msv332)
Supplement: Supplementary Data [file supp_33_4_946__index.html]

Long-Distance Dispersal Shaped Patterns of Human Genetic Diversity in Eurasia — Long-Distance Dispersal Shaped Patterns of Human Genetic Diversity in Eurasia — Supplementary Data 

# Long-Distance Dispersal Shaped Patterns of Human Genetic Diversity in Eurasia

## Supplementary Data

files

- Supplementary Data - pdf file
