# Supplementary material for: Long-Distance Dispersal Shaped Patterns of Human Genetic Diversity in Eurasia
Source: Mol Biol Evol. 2015 Dec 4;33(4):946–58. doi: 10.1093/molbev/msv332 (PMC4776706; doi:10.1093/molbev/msv332)
Supplement: Supplementary Data [file supp_msv332_revisions_MBE-15-0500.pdf]

# Supplementary Material

## Long distance dispersal shaped patterns of human genetic diversity in Eurasia

Isabel Alves, Miguel Arenas, Mathias Currat, Anna Sramkova Hanulova, Vitor C. Sousa, Nicolas Ray, Laurent Excoffier

## Supplementary Figures

### Supplementary Figure 1

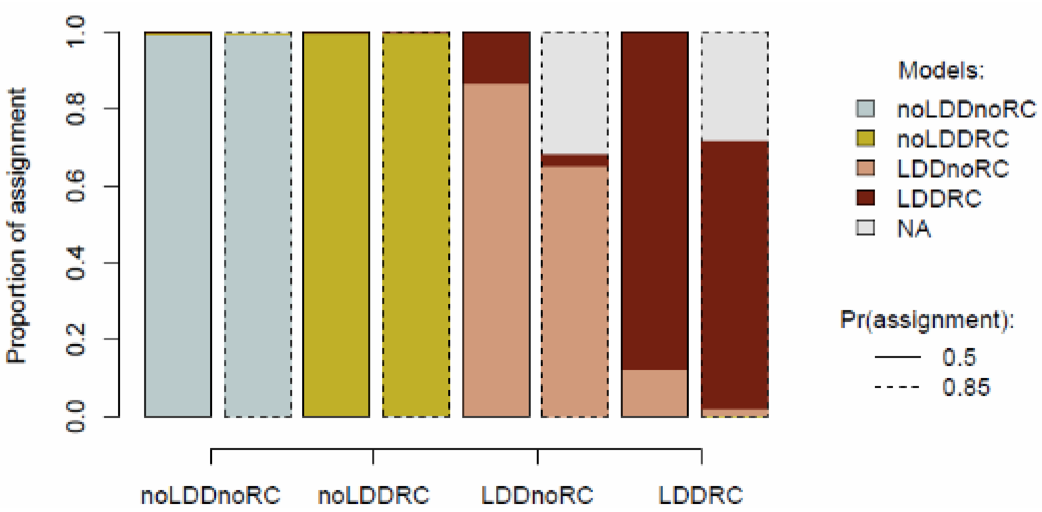


**Figure S1.** Validation of the four main evolutionary models. Model posterior probabilities were computed using the multivariate logistic regression (Beaumont 2008). Colors within the bars are proportional to the number of PODS simulated under the models on the x-axis and assigned to any of the models represented on the right-side of the picture. Two thresholds of 0.50 (solid line bars) and 0.85 (dashed line bars) were used to assign a PODS to a given model. NA stands for those PODS that could not be assigned. 1,000 PODS and 99,000 simulations per model were used to perform model validation (see Material and Methods for details and supplementary Table S1 for exact proportions).

## Supplementary Figure 2

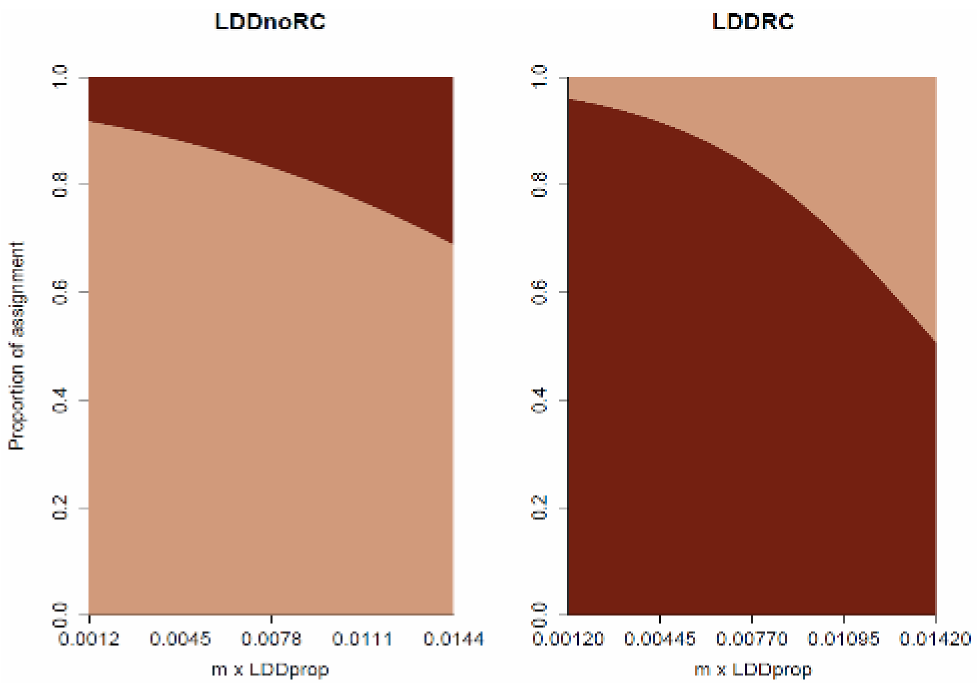


**Figure S2.** Proportion of correct model assignment as a function of the product of LDD proportion (LDDprop) and migration rate (m), referred to hereafter as effective LDD. The range of values of effective LDD found within the 1,000 PODS (generated under each model) was divided into bins. PODS were allocated to their corresponding bin and a binomial logistic regression was applied. Note that the proportions of PODS allocated to the right model were computed assuming a posterior probability 0.50. The *LDDnoRC* and the *LDDRC* models are represented in salmon and dark red, as shown previously in supplementary Fig. S1.

## Supplementary Figure 3A

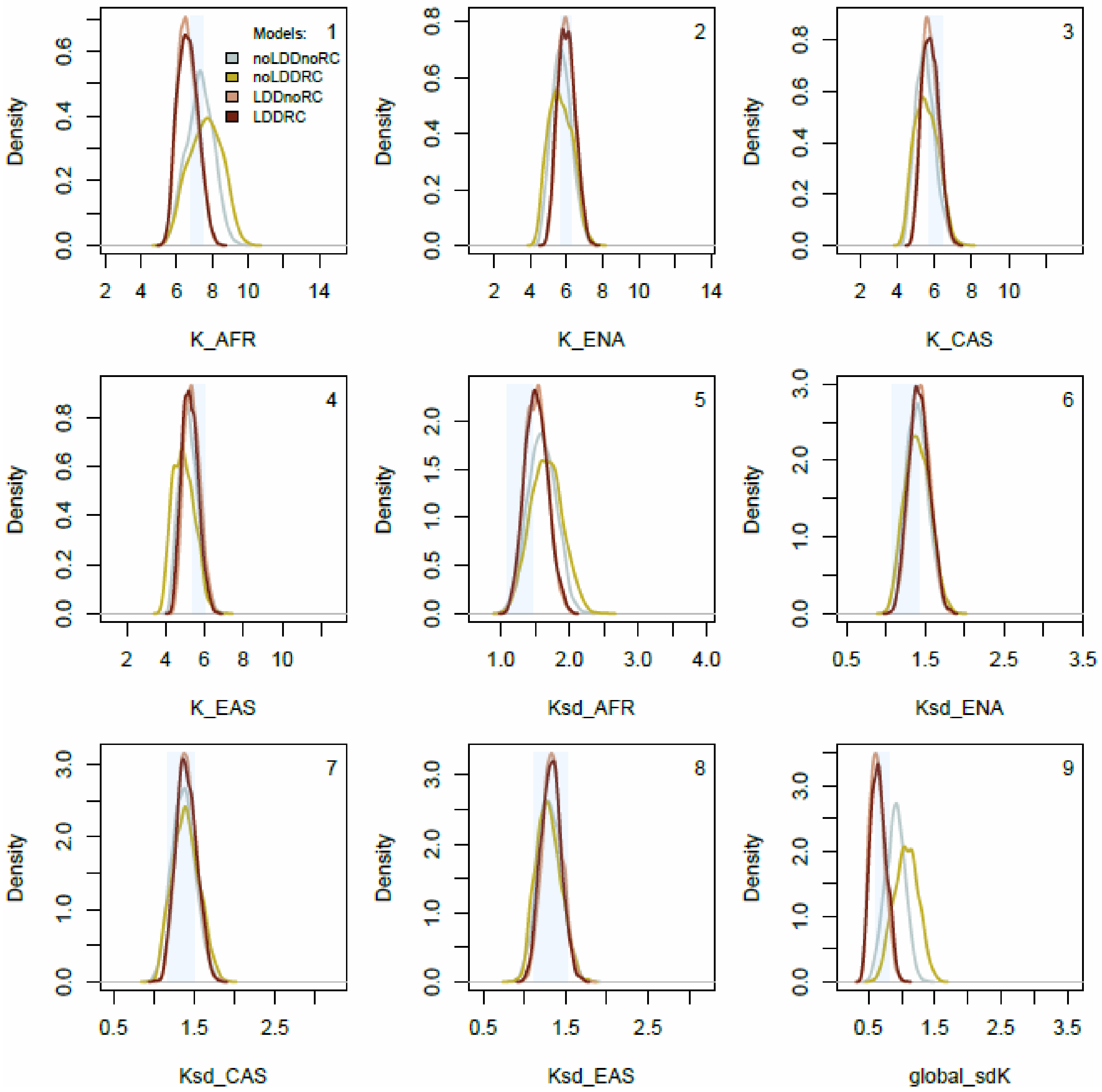

**Supplementary Figure 3A cont.**

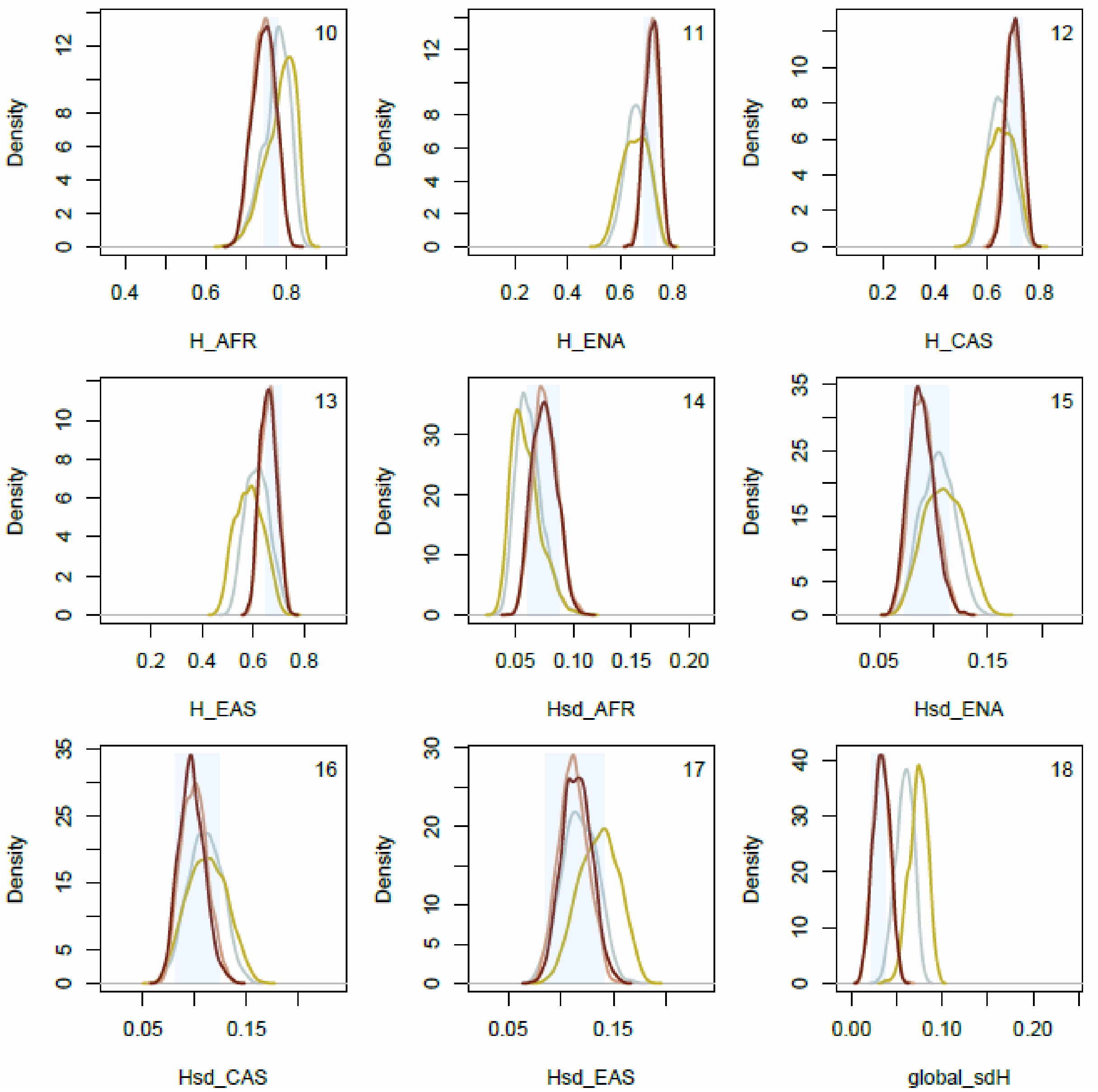

**Supplementary Figure 3A cont.**

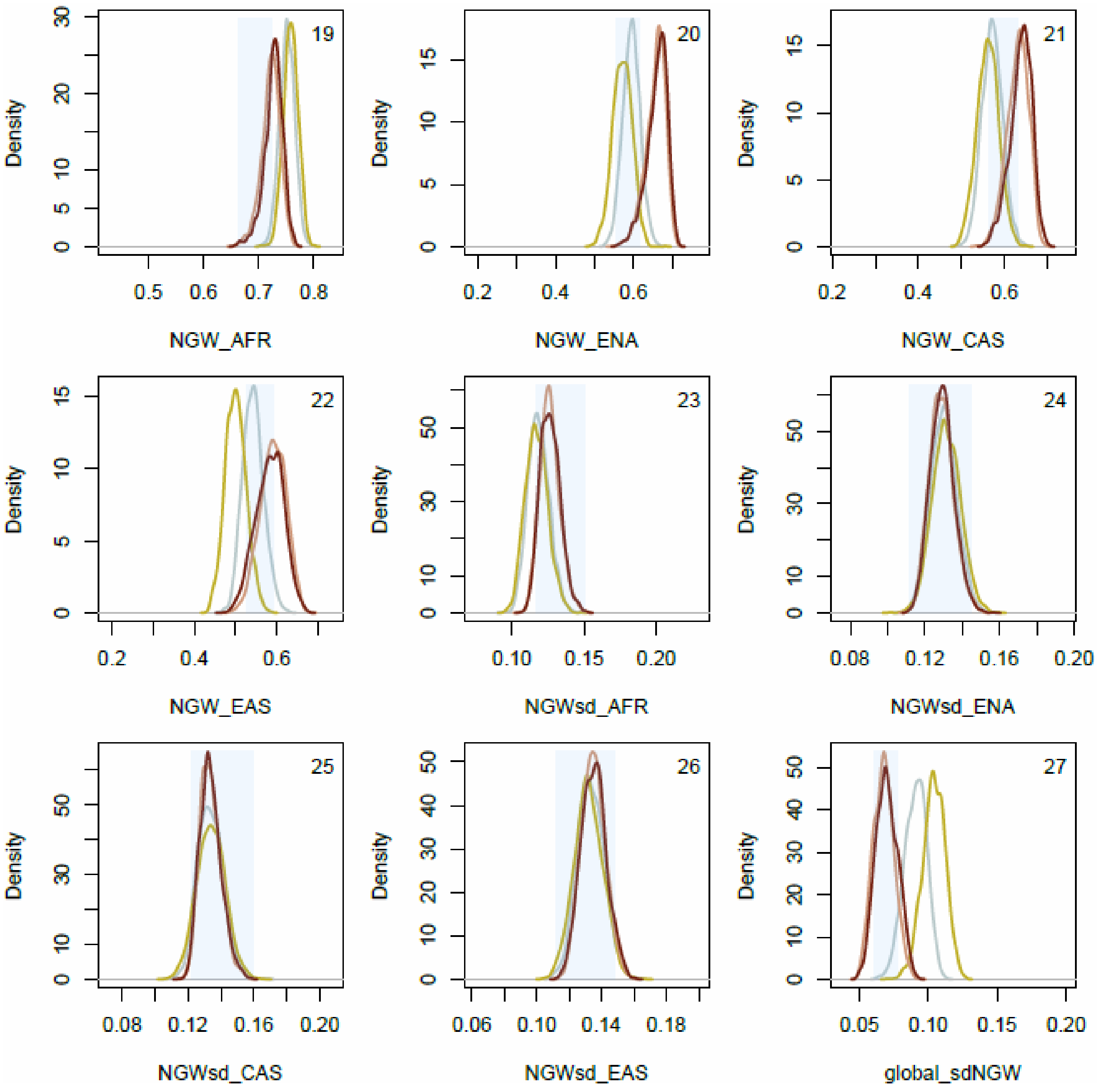

**Supplementary Figure 3A cont.**

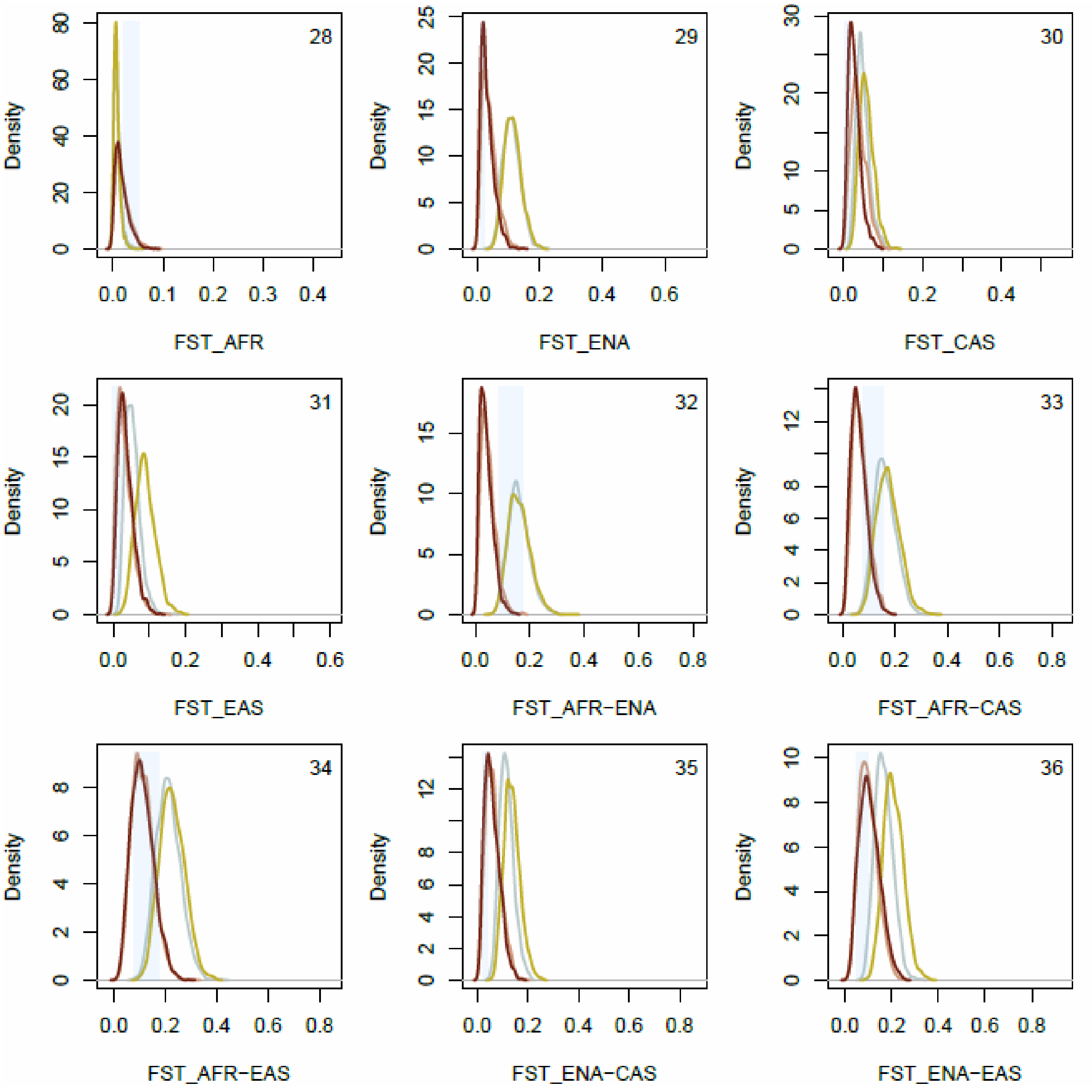

**Supplementary Figure 3A cont.**

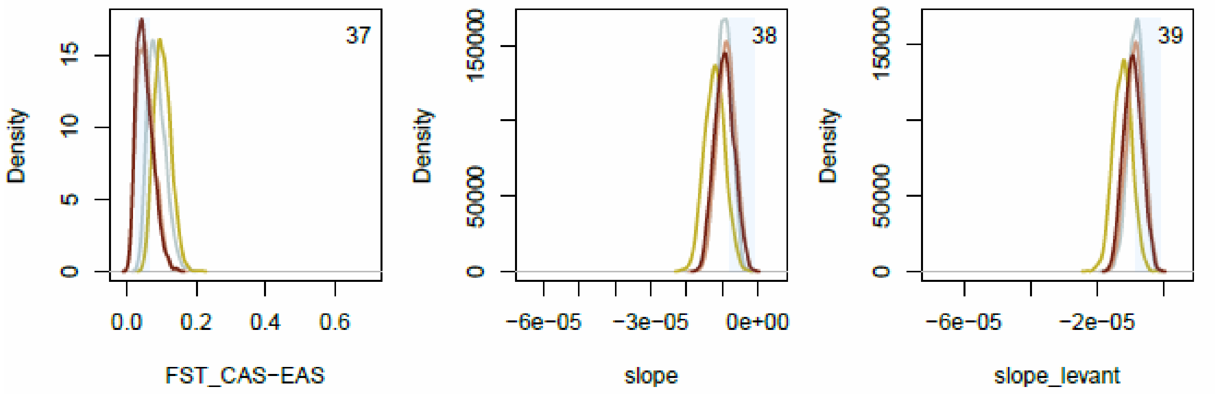


**Figure S3A.** Distributions of the summary statistics in the 2% simulations closest to the observed data generated under the *noLDDnoRC*, *noLDDRC*, *LDDnoRC* and *LDDRC*. Shaded area represents the distribution of values of each SS among the 1,000 bootstrap observed datasets. SS are fully described in Table S4. The x-axis corresponds to the prior range of the summary statistics.

**Supplementary Figure 3B**

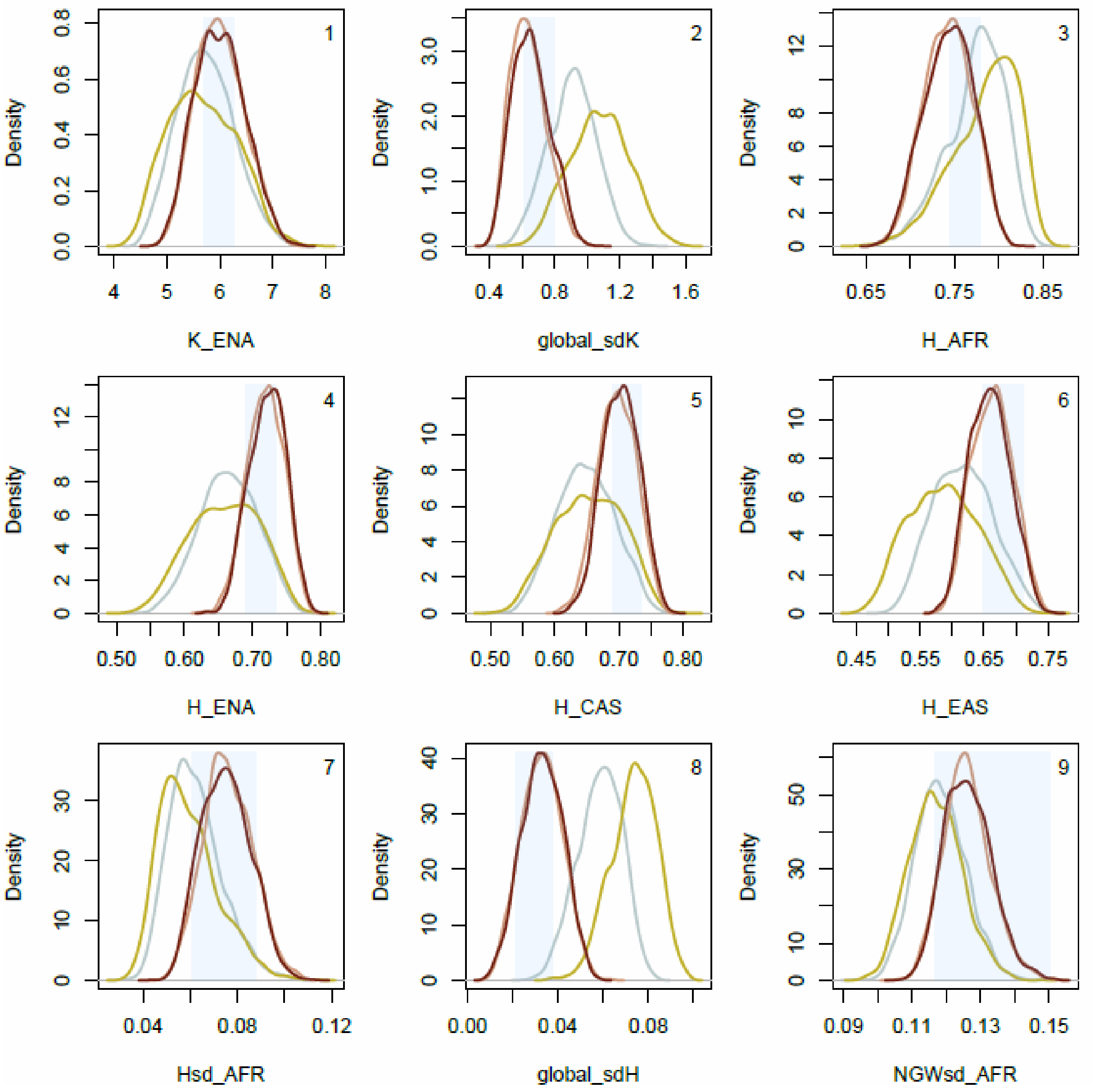

**Supplementary Figure 3B cont.**

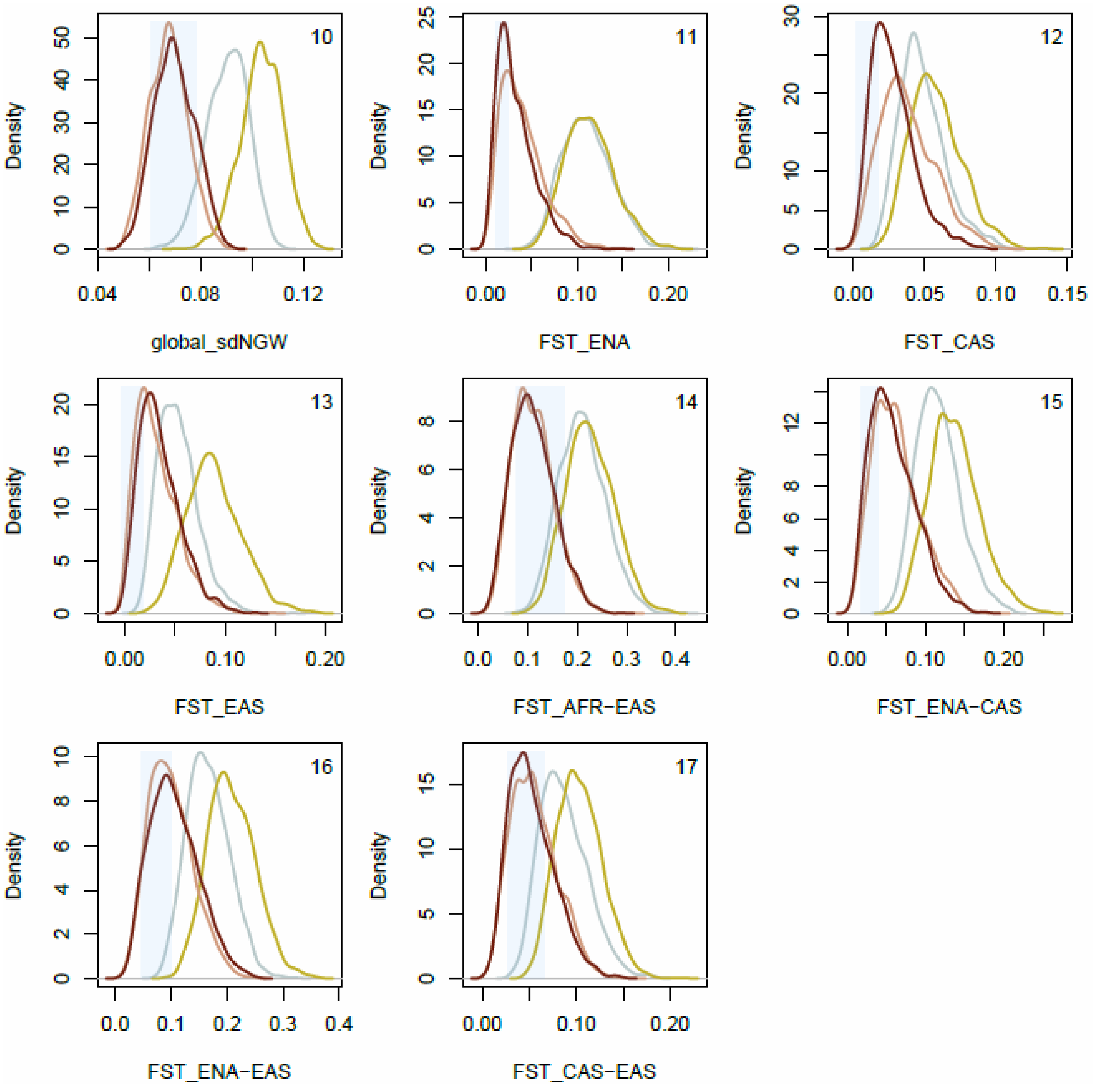


**Figure S3B.** Distribution of the summary statistics supporting LDD in the 2% best simulations (closest to the observed data) generated under the four classical models. Color code is the same as in supplementary Fig. S3A. Shaded area represents the distribution of values of each SS among the 1,000 bootstrap observed datasets. SS are fully described in Table S4. The x-axis corresponds to the whole range of the summary statistics in the 2% best simulations of each model.

## Supplementary Figure 3C

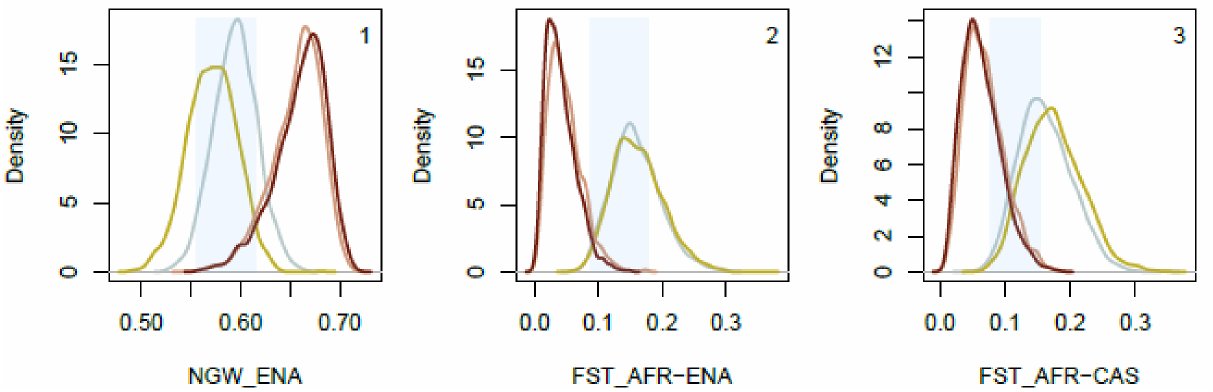


**Figure S3C.** Distribution of the summary statistics irreproducible by LDD models in the 2% best simulations generated under the four classical models. Color code is the same as in supplementary Fig. S3A. Shaded area represents the distribution of values of each SS among the 1,000 bootstrap observed datasets. SS are fully described in Table S4. The x-axis corresponds to the whole range of the summary statistics in the 2% best simulations of each model.

## Supplementary Figure 4

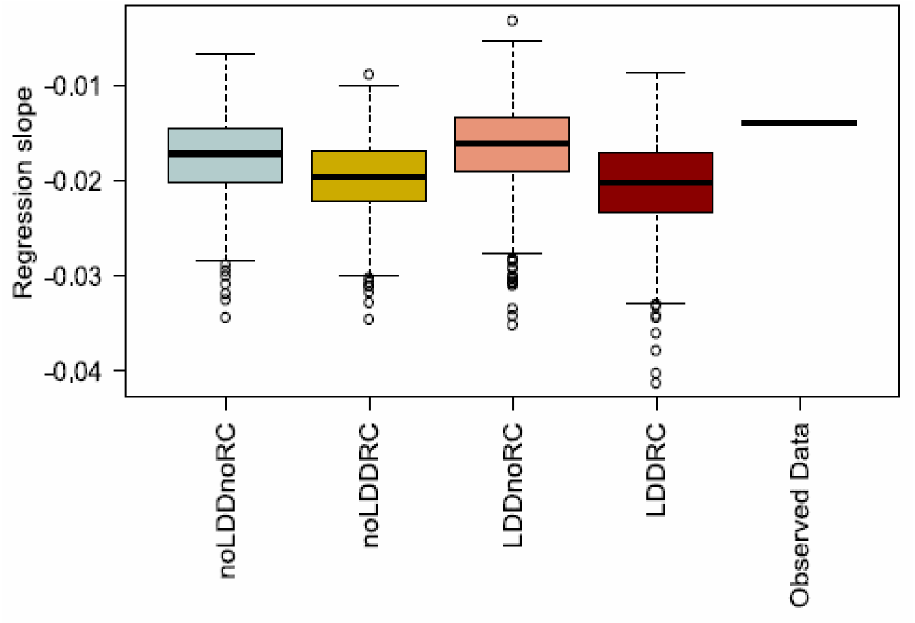


**Figure S4.** Distributions of regression slopes obtained from simulated and observed data. We simulated 1,000 datasets under different models (x-axis), drawing parameters from the parameter posterior distributions, computed population-specific number of alleles and applied a linear regression using the corresponding population latitude as an explanatory variable. We applied the same approach to the 1,000 observed bootstrap replicates (rightmost column), which is shown as a simple horizontal line due to the low variance among replicates.

## Supplementary Figure 5

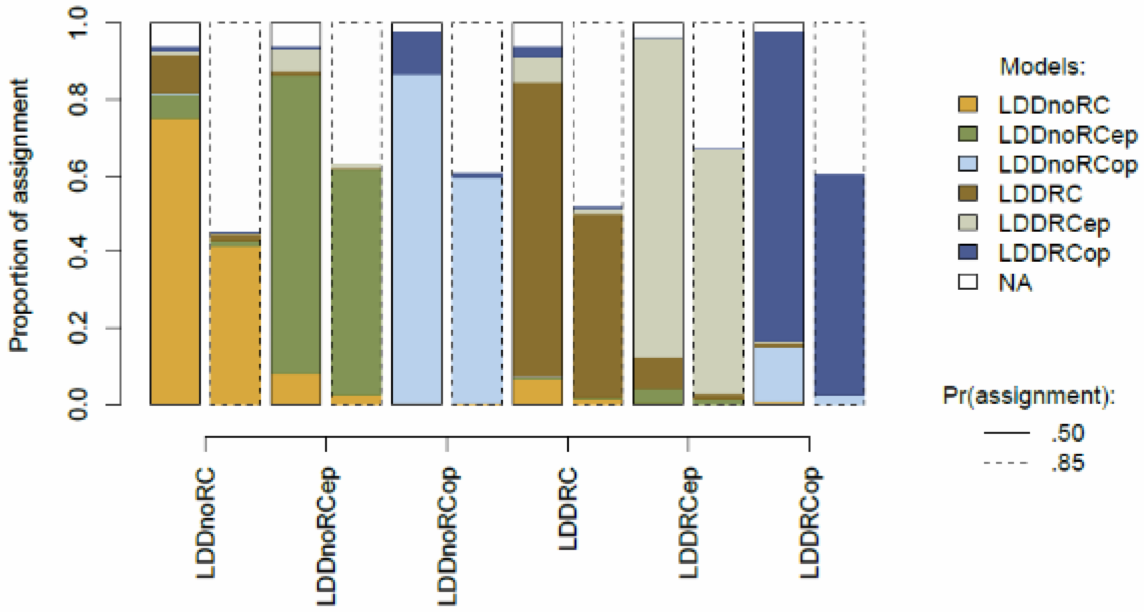


**Figure S5.** Model validation of the six models involving LDD using the multivariate logistic regression (Beaumont 2008). Colors within the bars are proportional to the number of PODS simulated under the models on the x-axis and assigned to any of the models represented on the right-side of the picture. Two thresholds were used to assign a PODS to a given model: 0.50 (solid contour lines) and 0.85 (dashed contour lines). NA stands for those PODS that could not be assigned. 1,000 PODS and 19,000 simulations per model were used to perform model validation (see Material and Methods for details, and supplementary Table S2 for exact proportions).

**Supplementary Figure 6A**

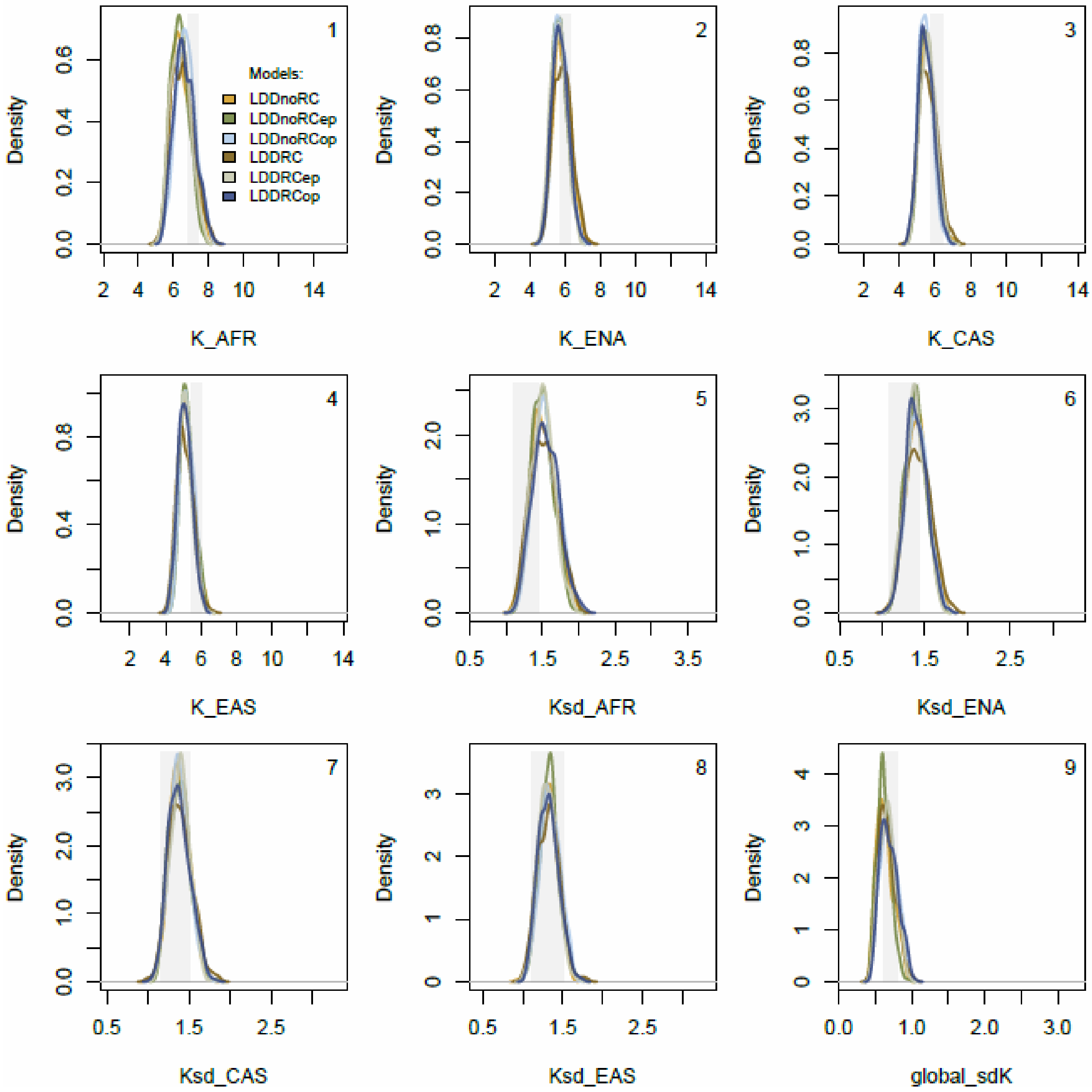

**Supplementary Figure 6A cont.**

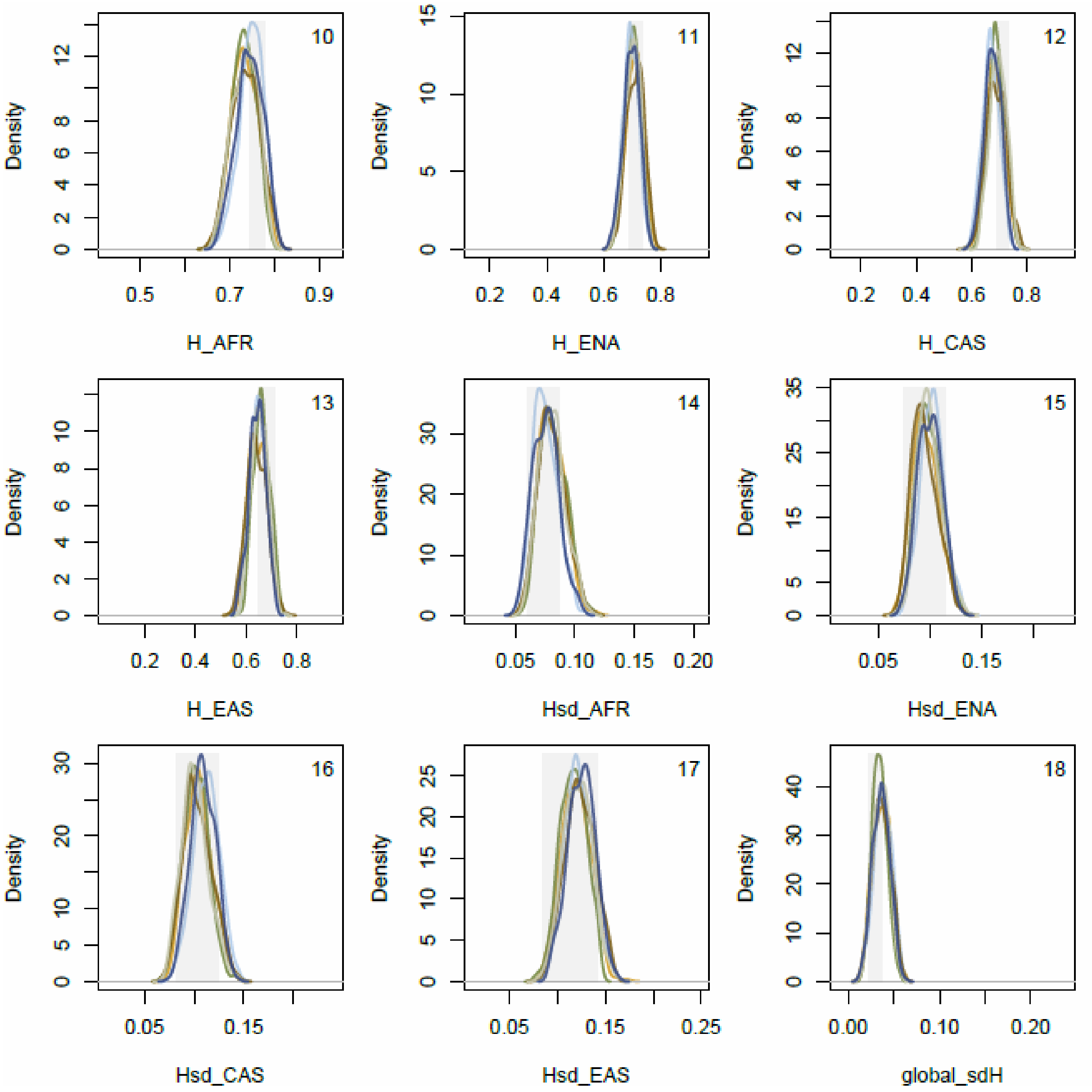

**Supplementary Figure 6A cont.**

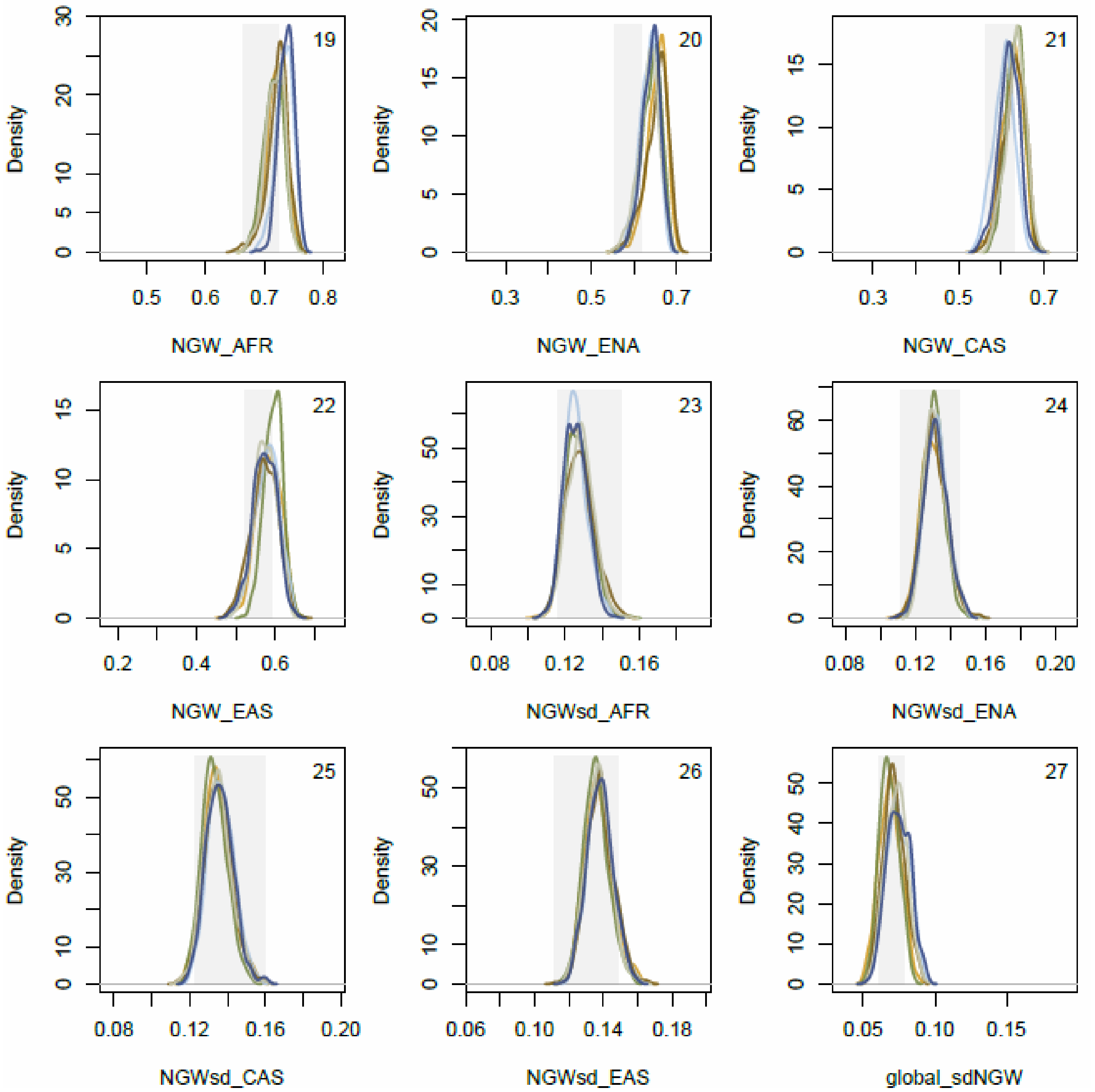

**Supplementary Figure 6A cont.**

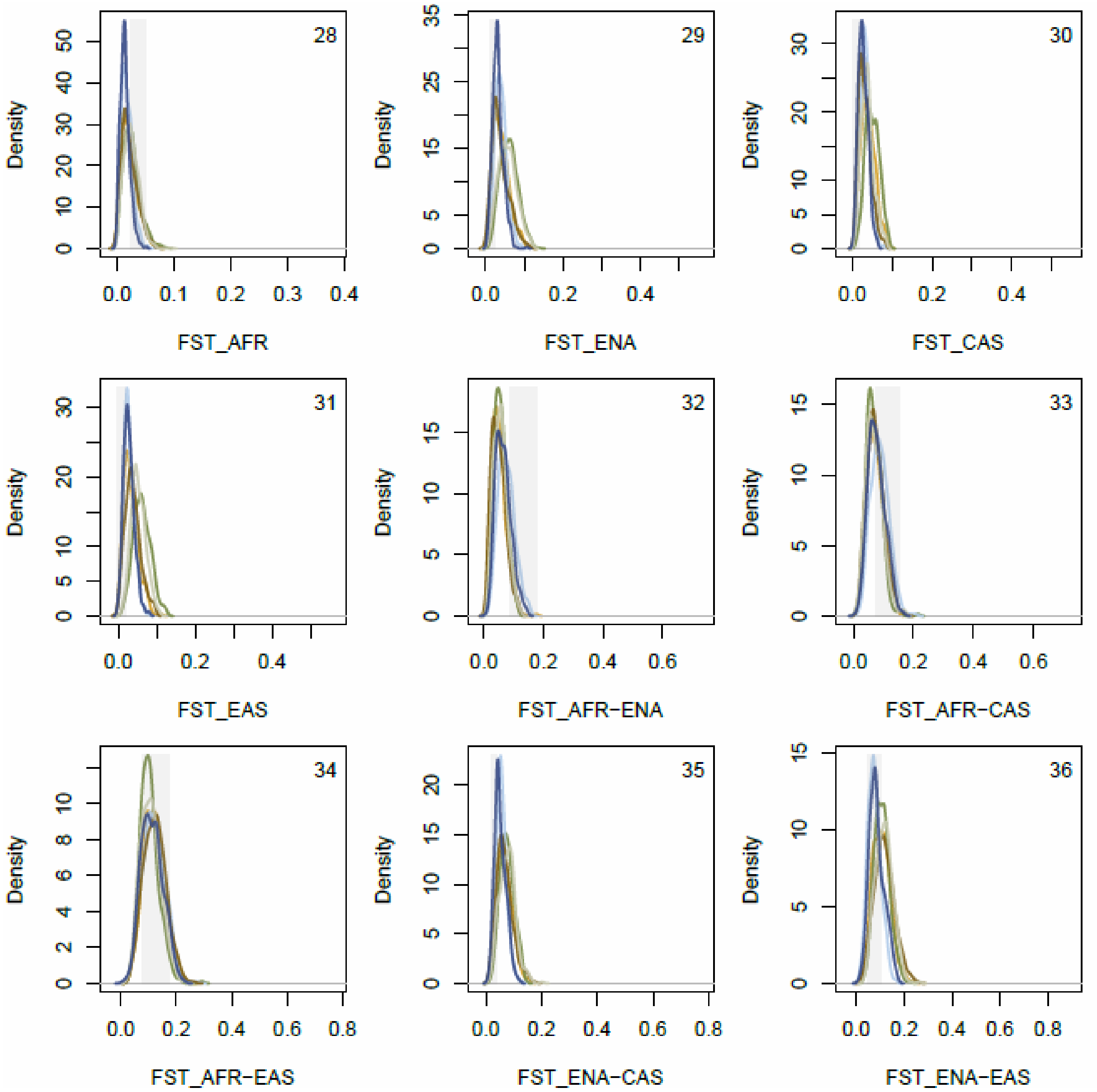

**Supplementary Figure 6A cont.**

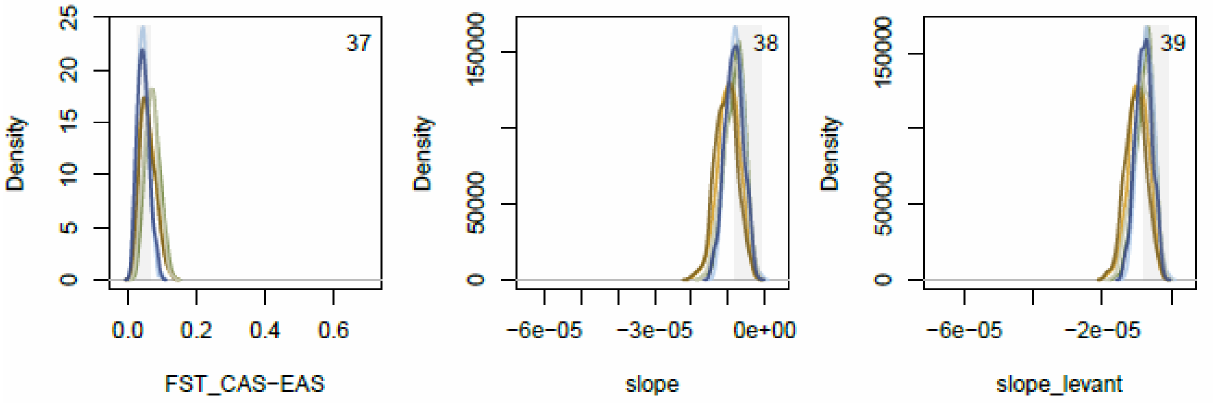


**Figure S6A.** Distributions of summary statistics in the 10% best simulations (closest to the observed data) under each of the six models involving long distance dispersal. Shaded area represents the distribution of values of each SS among the 1,000 bootstrap observed datasets. SS are fully described in Table S4. The x-axis corresponds to the prior range of the summary statistics.

## Supplementary Figure 6B

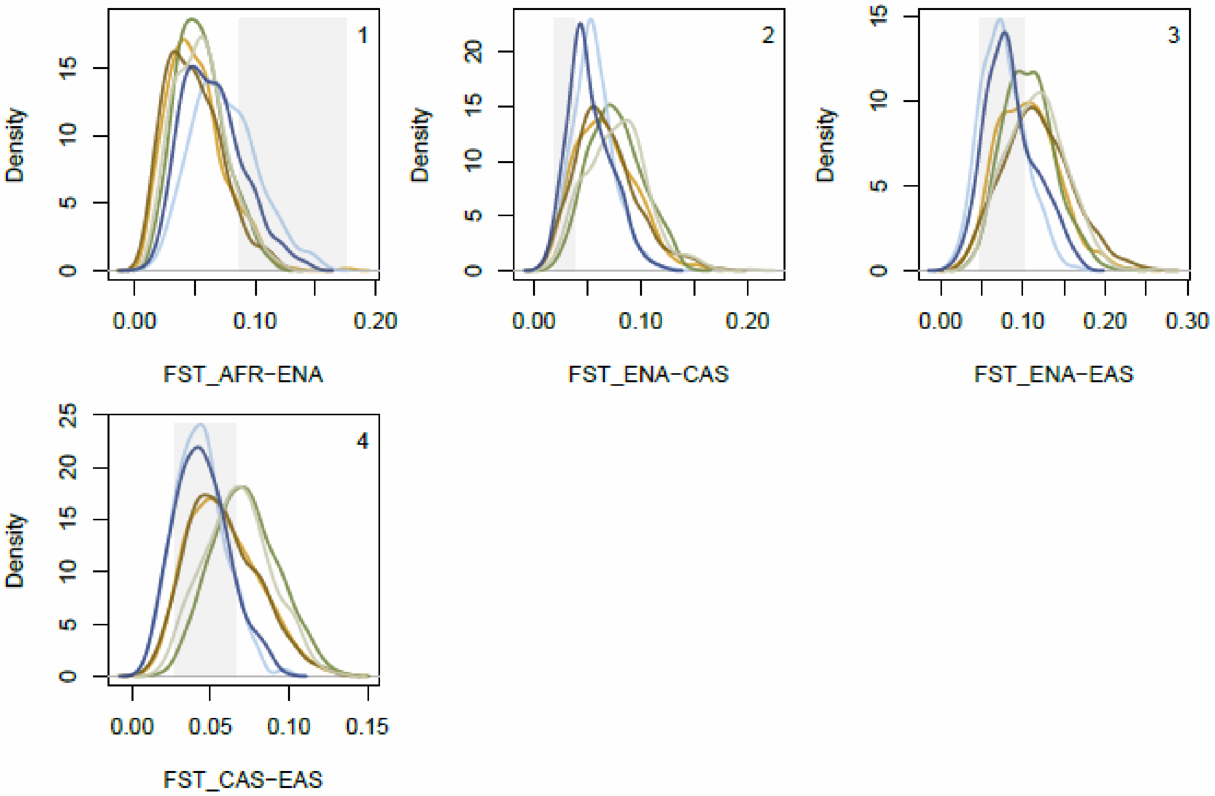


**Figure S6B.** Distributions of most discriminant summary statistics in the 10% best simulations (closest to the observed data) among the six models involving long distance dispersal. Color code is the same as in supplementary Fig. S6A. Shaded area represents the distribution of values of each SS among the 1,000 bootstrap observed datasets. SS are fully described in Table S4. The x-axis corresponds to the range of values for the summary statistics in the 10% best simulations of each model.

**Supplementary Figure 7**

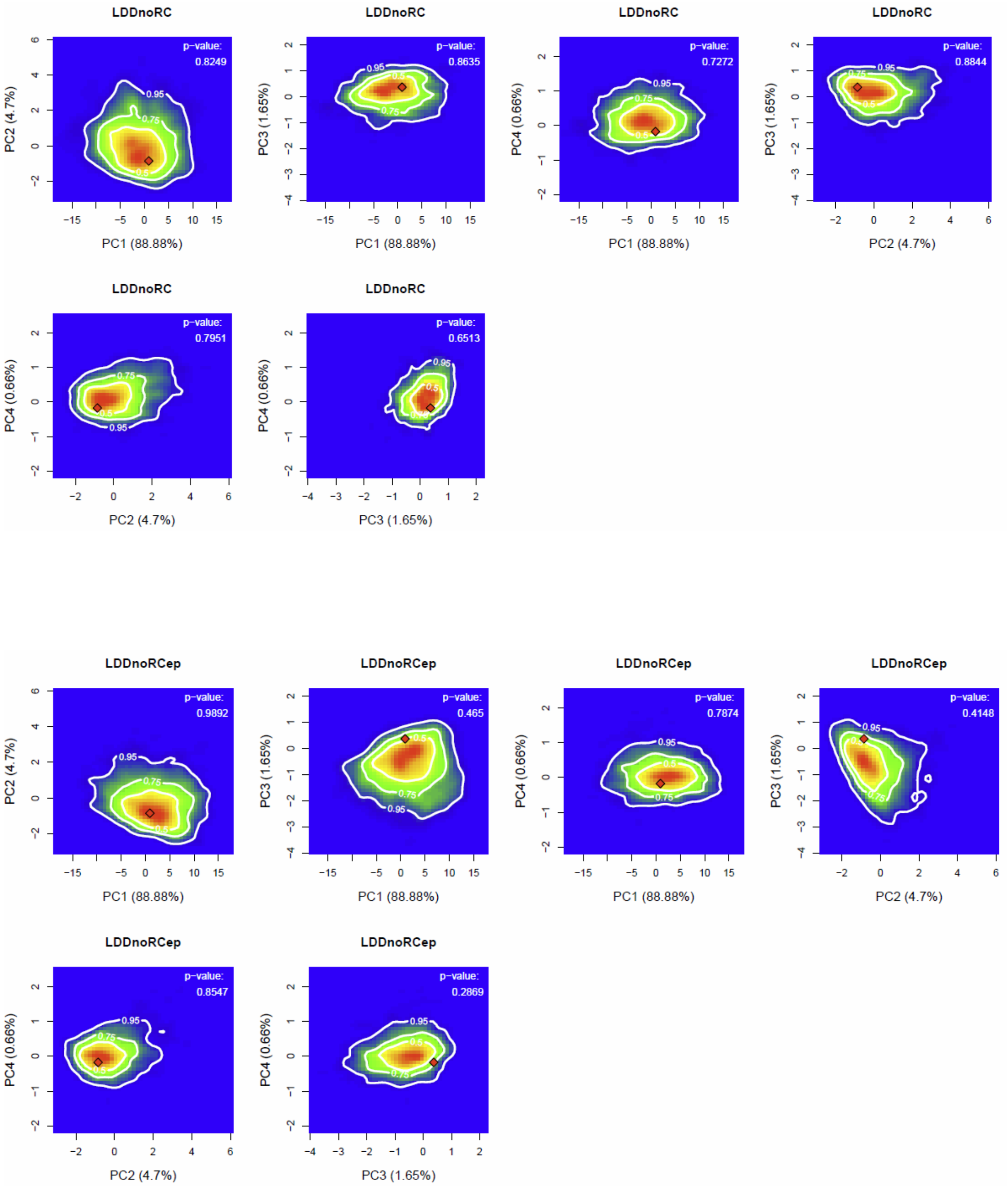

**Supplementary Figure 7 cont.**

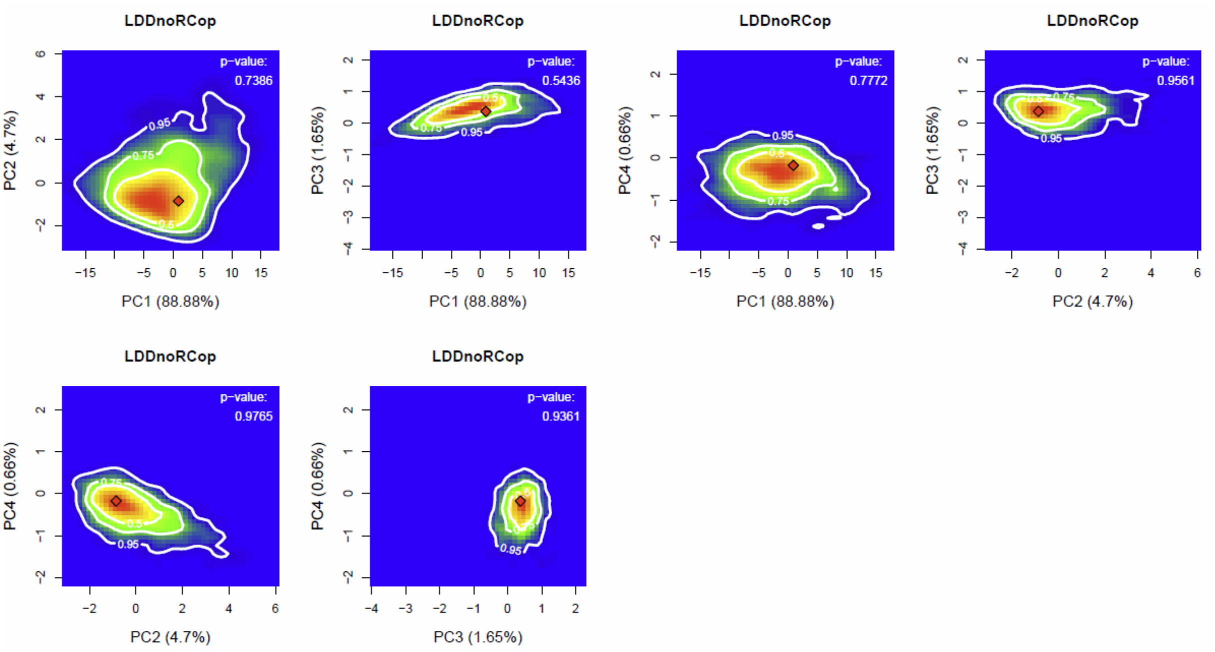


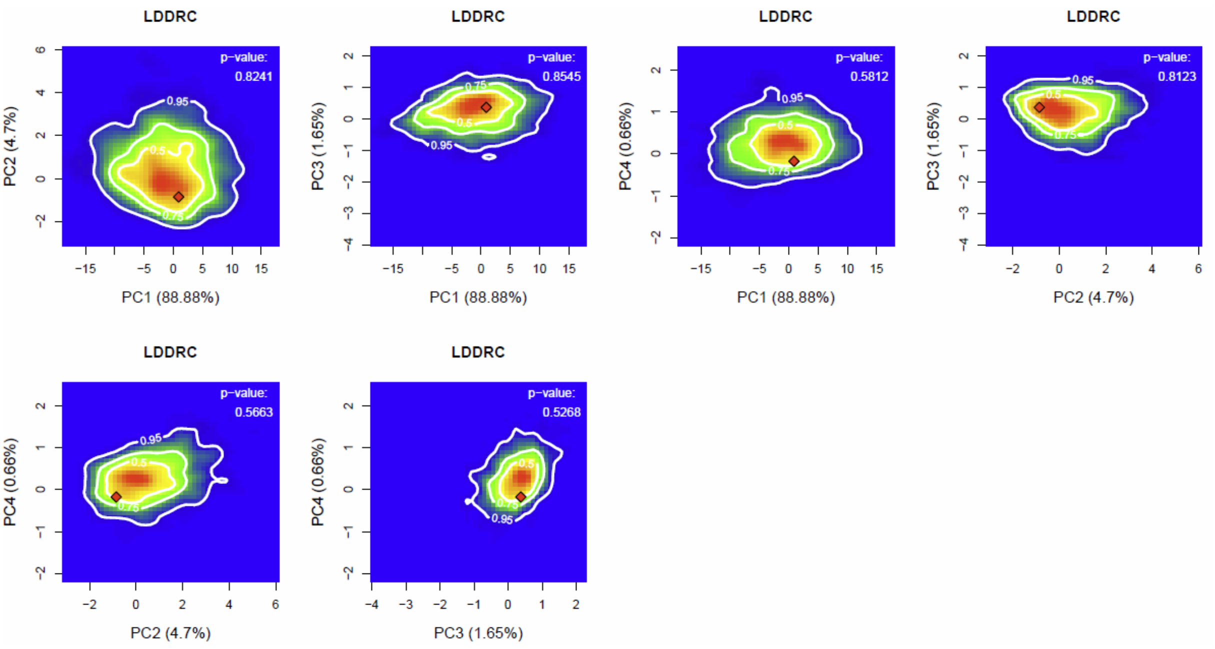

**Supplementary Figure 7 cont.**

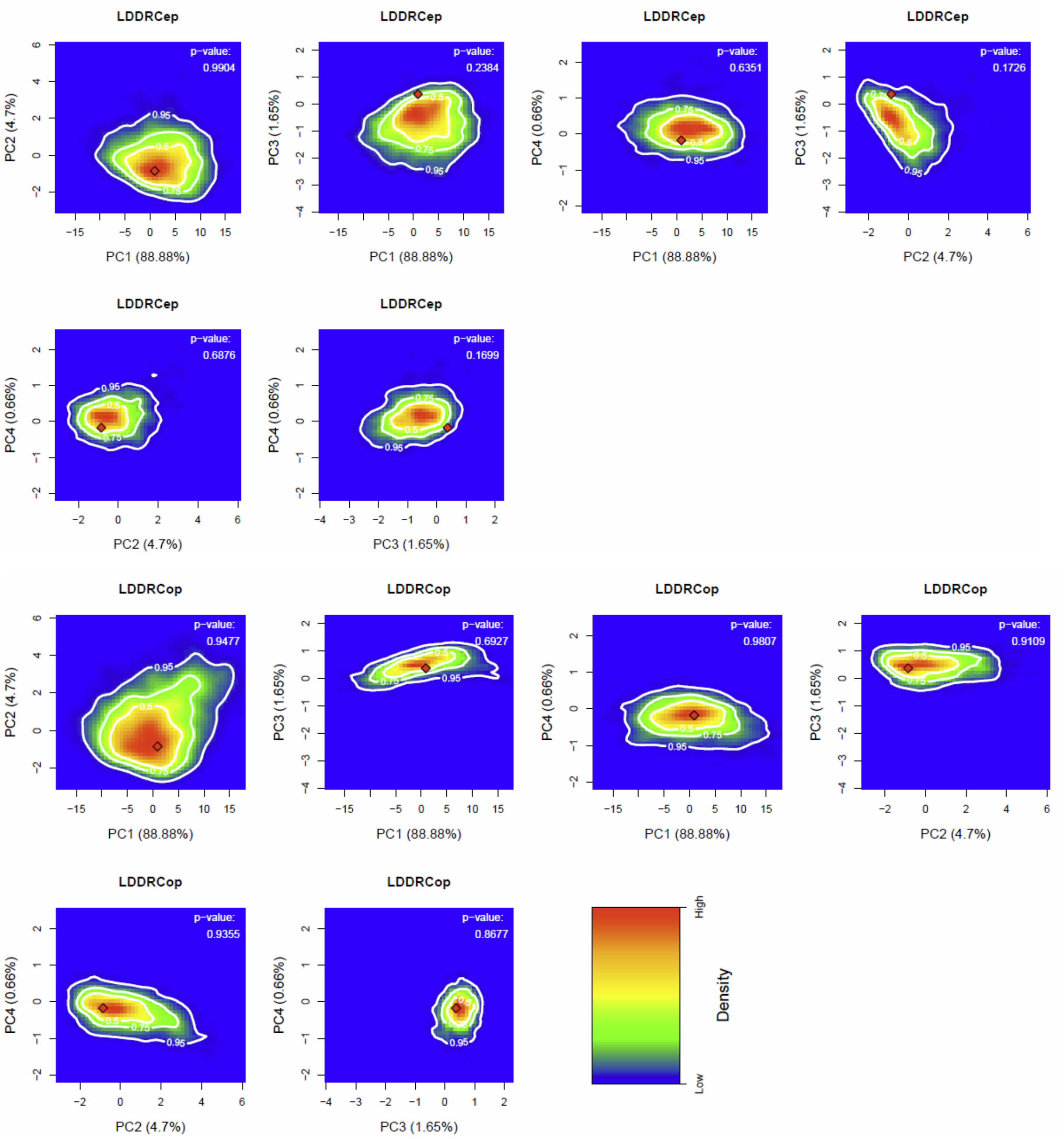


**Figure S7.** Principal Component Analysis of the summary statistics generated from the posterior distributions of each of the six models involving LDD. The distribution of the pairwise combination of the first four PCs is shown together with the PC coordinates from the observed summary statistics (red diamond). White lines correspond to the 50%, 75% and 95% distribution quantiles. To represent the real data (red diamond) we chose the resampled dataset (out of 1,000) with a model posterior probability in favor of the model *LDDRCop* (best model) corresponding to the median of the

distribution. 2D P-values were computed as described in (Daub, et al. 2014) and are shown in the bottom left corner of each plot.

## Supplementary Figure 8

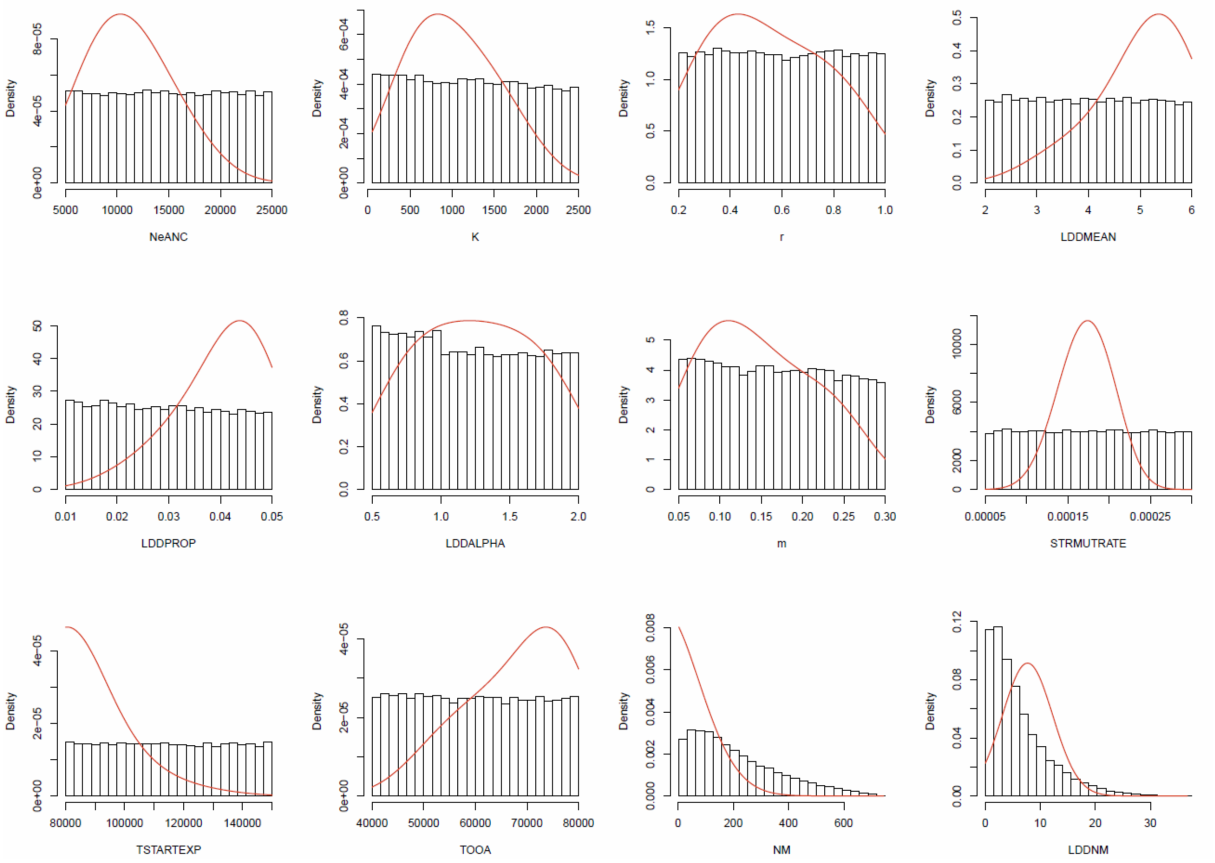


**Figure S8.** Posterior distributions of the parameters of the best model *LDDRCop*. The prior distributions are shown as a histogram (Table S3) and parameter posterior densities are represented by the red line. We used a total of 50,000 simulations, retained the best 2,000 (closest to the observed data) and used a general linear model (GLM) to approximate the likelihood function.

**Supplementary Figure 9**

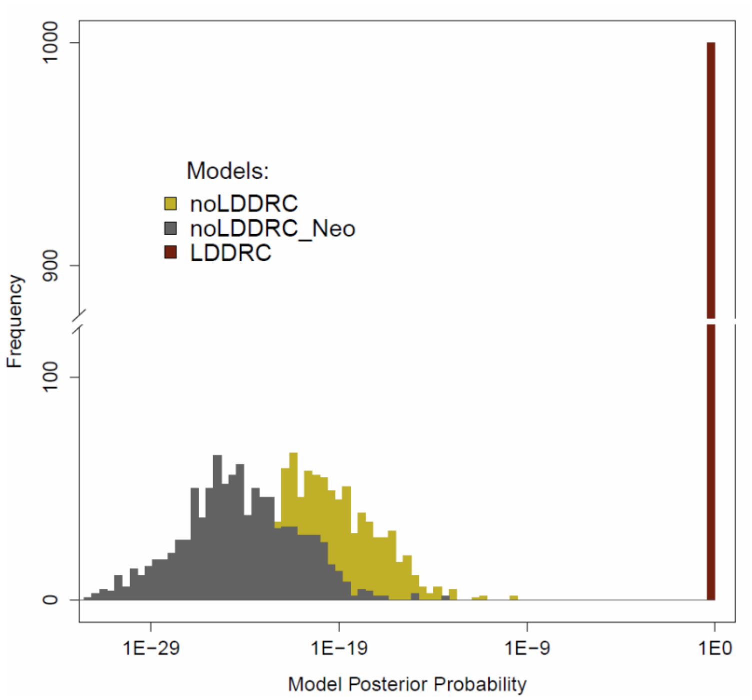


**Figure S9.** Comparison of posterior probabilities of scenarios involving range contractions, and LDD or Neolithic expansions. Posterior probabilities for these scenarios were obtained for 1,000 bootstrap datasets by applying the multivariate logistic regression (Beaumont 2008) on the 2% best simulations among 50,000 simulations per evolutionary scenario.

**Supplementary Figure 10**

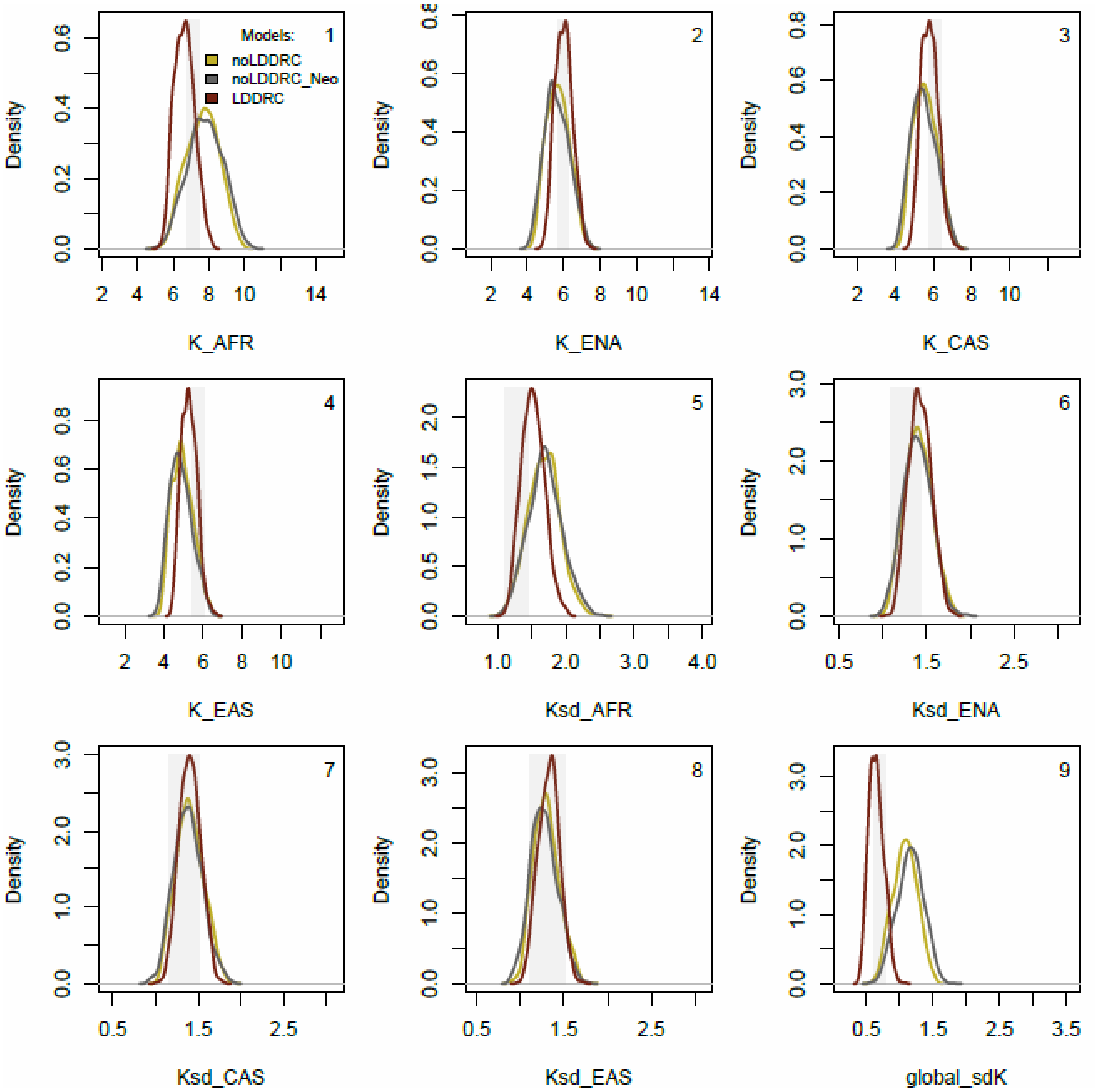

**Supplementary Figure 10 cont.**

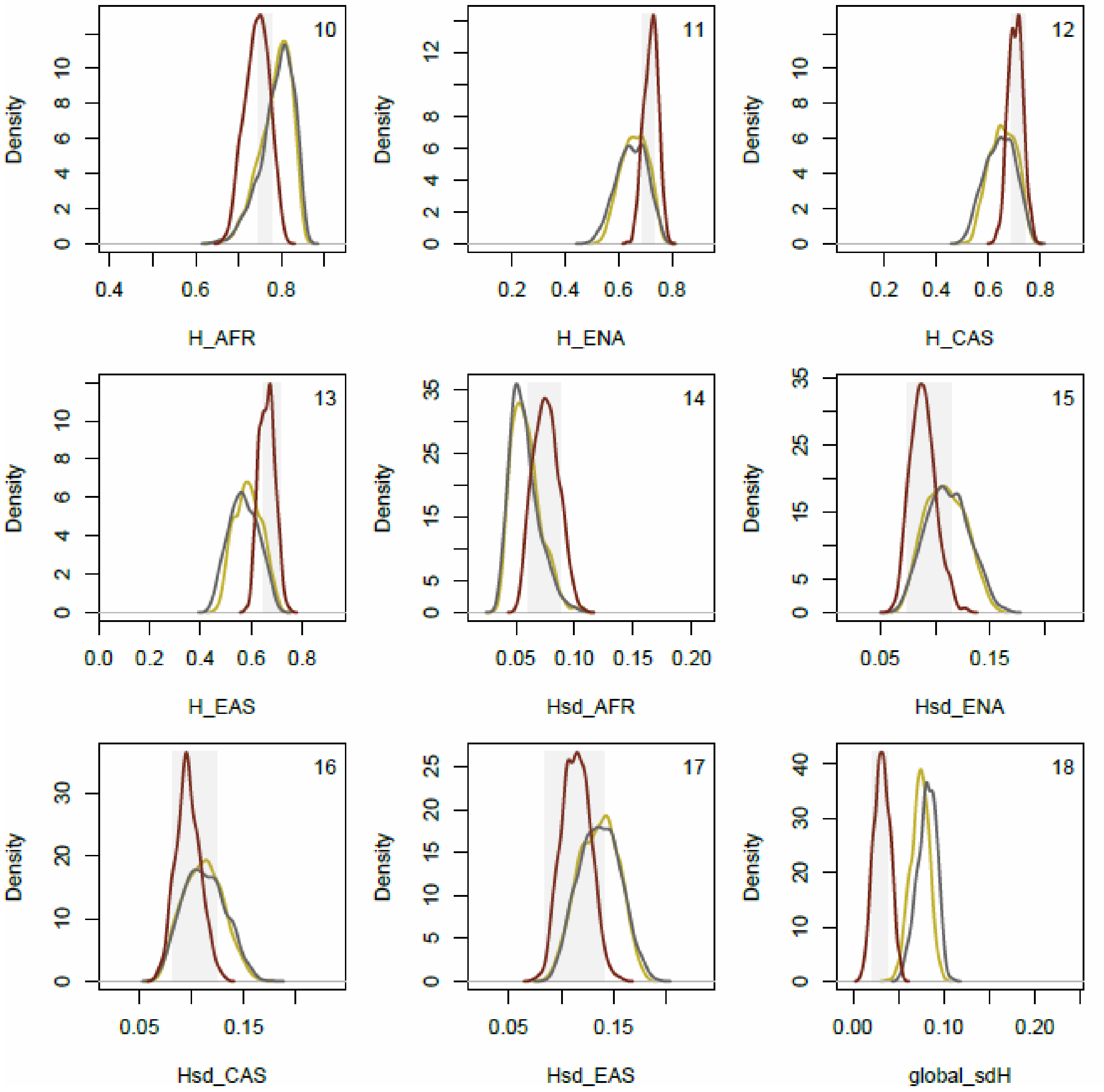

**Supplementary Figure 10 cont.**

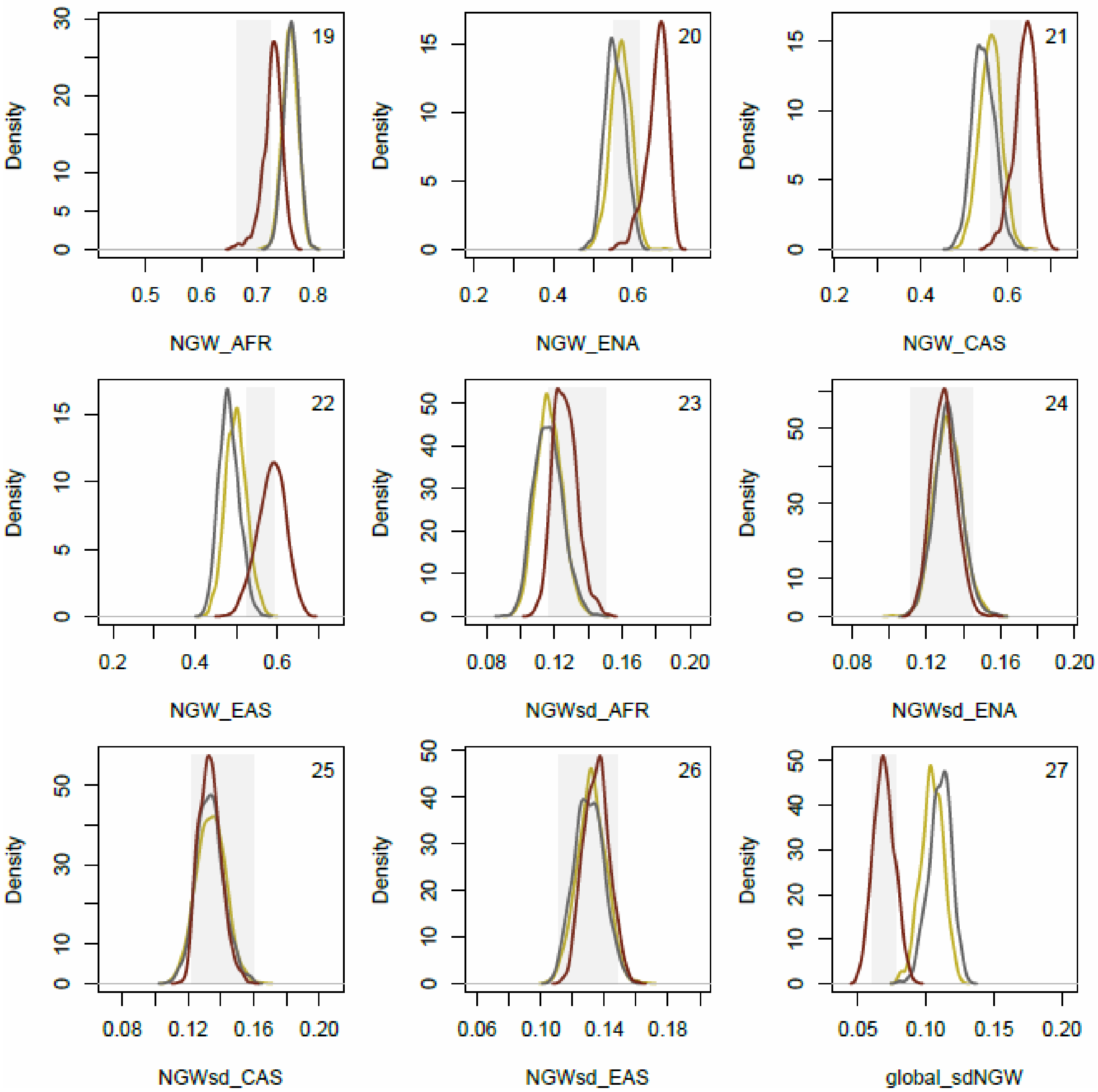

**Supplementary Figure 10 cont.**

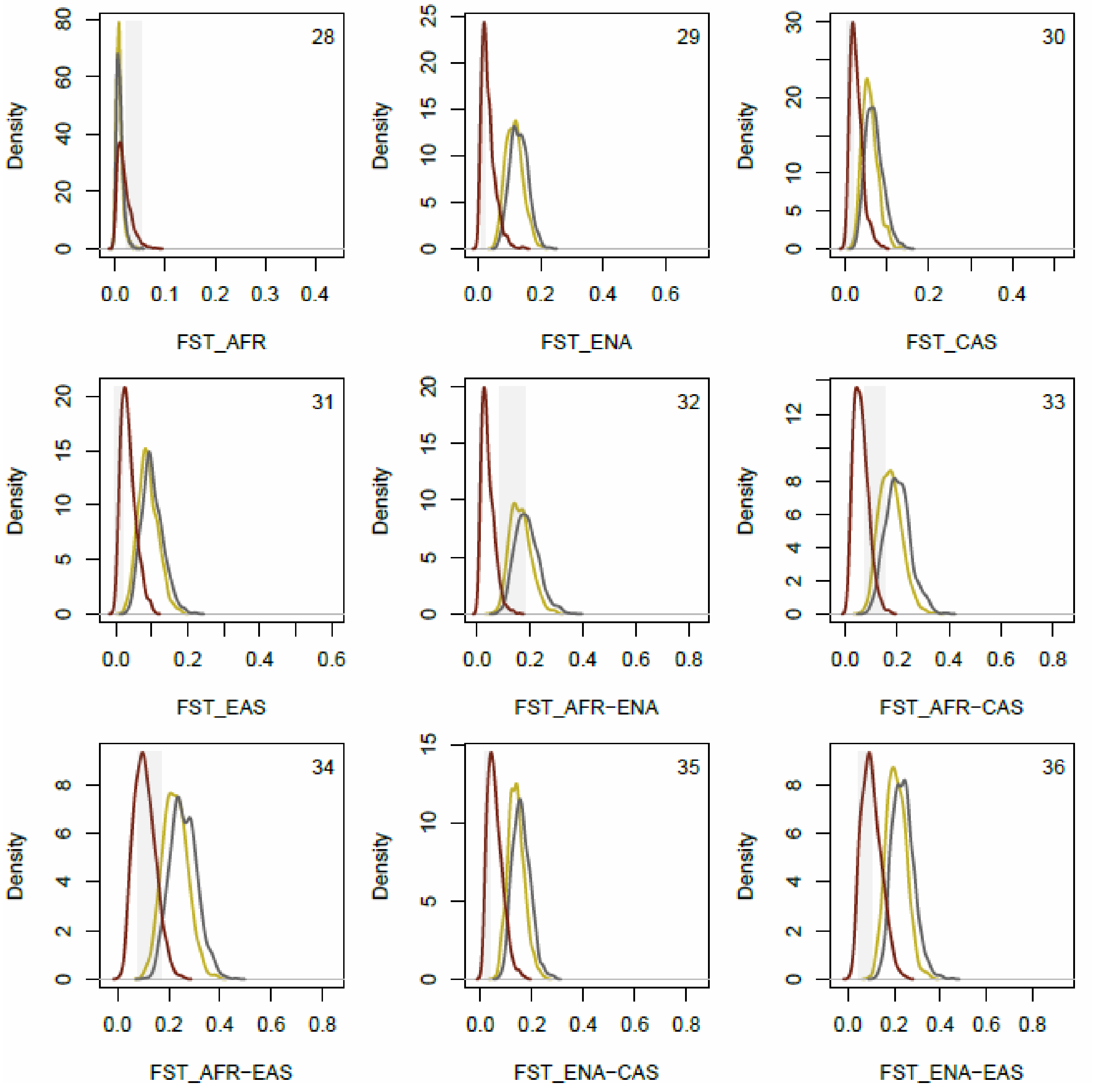

**Supplementary Figure 10 cont.**

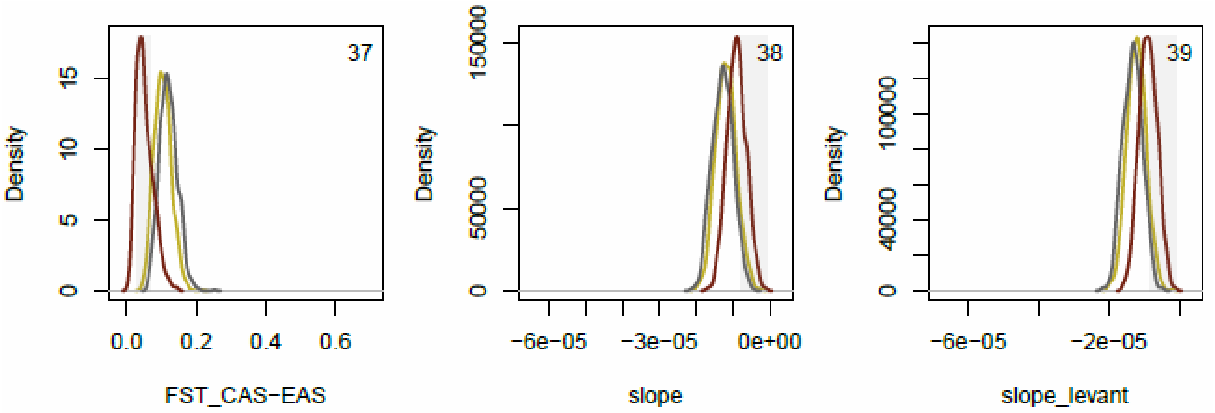


**Figure S10.** Distributions of summary statistics in the 2% best simulations under a model with LDD events and a range contraction, and two other models without LDD but one including a Neolithic population size increase. Shaded area represents the distribution of values of each SS among the 1,000 resampled observed datasets. SS are fully described in Table S4. The x-axis corresponds to the prior range of the summary statistics.

## Supplementary Figure 11

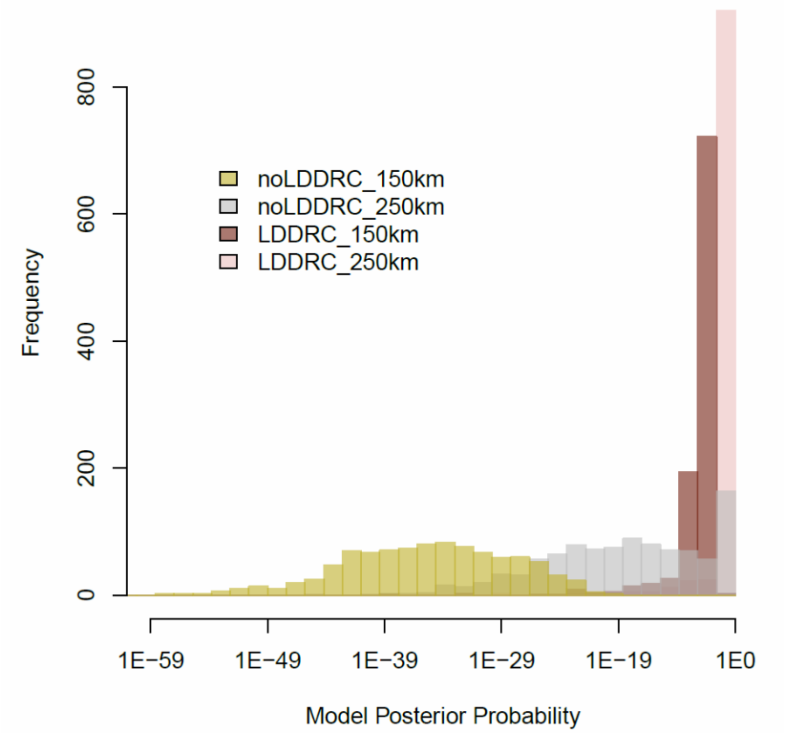


**Figure S11.** Distributions of the posterior probabilities of four scenarios of human expansions assuming different deme physical dimensions (*noLDDRC* 150x150km, *noLDDRC* 250x250km, *LDDRC* 150x150km, *LDDRC* 250x250km) obtained over the 1,000 bootstrap datasets. Model posterior probabilities were computed using the multivariate logistic regression (Beaumont 2008) on the 10% best simulations among 10,000 simulations per evolutionary scenario. Whereas the *LDDRC* model with larger deme sizes is the overall best model, we note that the *noLDDRC* model with larger deme sizes becomes the best-supported model for a few bootstrap replicates (121/1000). In line with these results, we find that most of the SS distributions generated under the *noLDDRC* model with large deme dimensions are now intermediate between that of the original *noLDDRC* model and those of models including LDD (supplementary Fig. S12).

**Supplementary Figure 12**

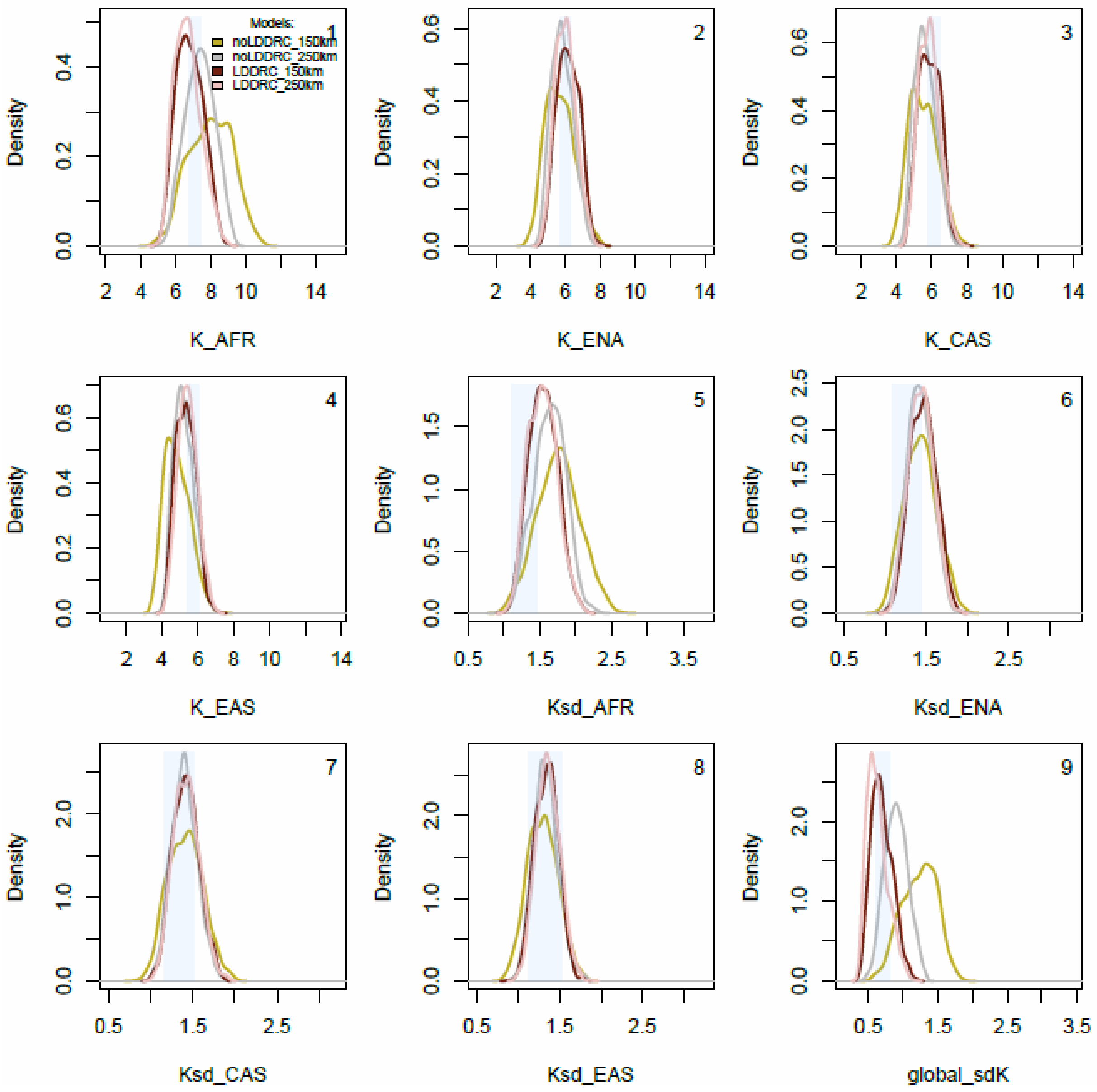

**Supplementary Figure 12 cont.**

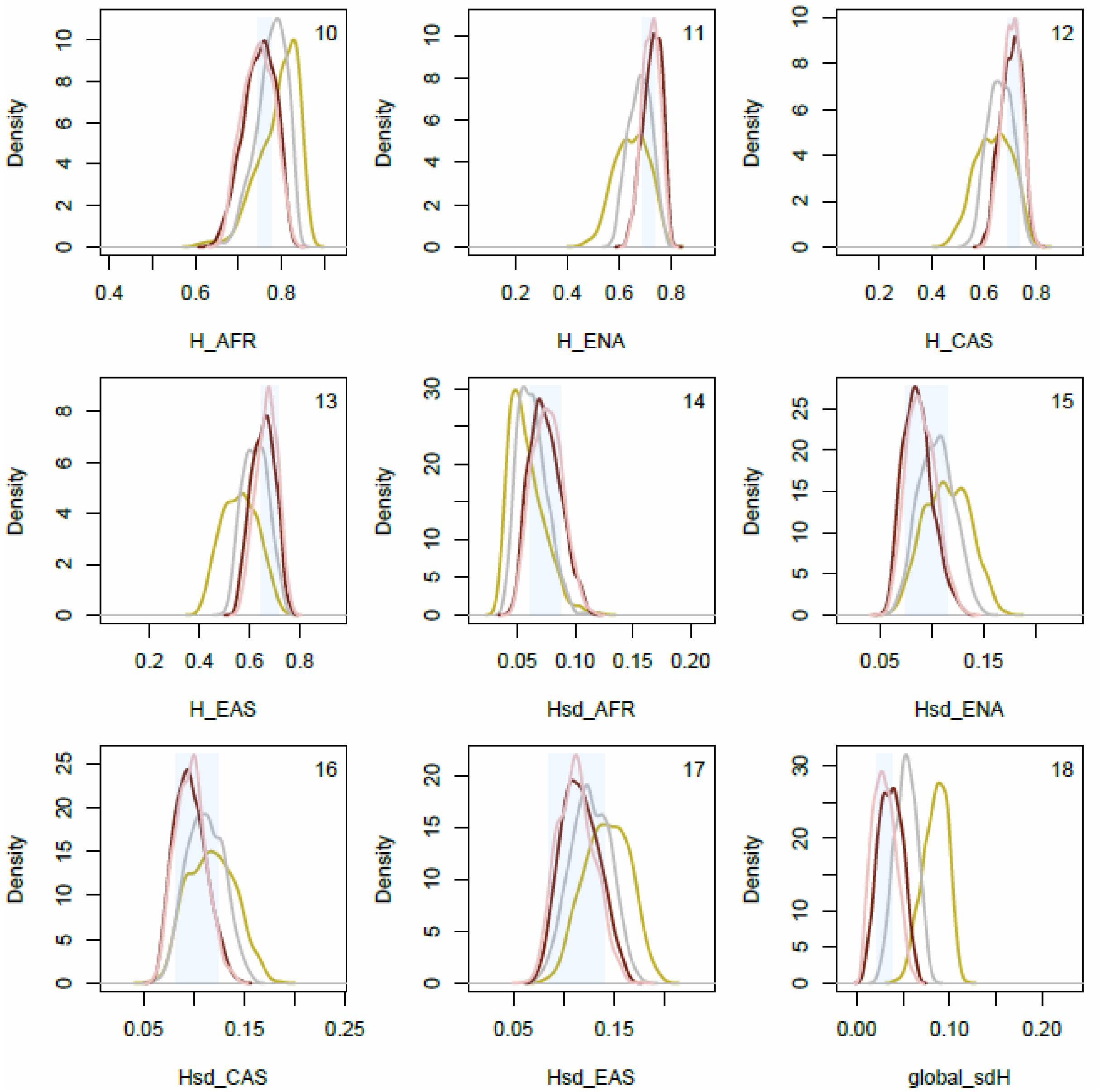

**Supplementary Figure 12 cont.**

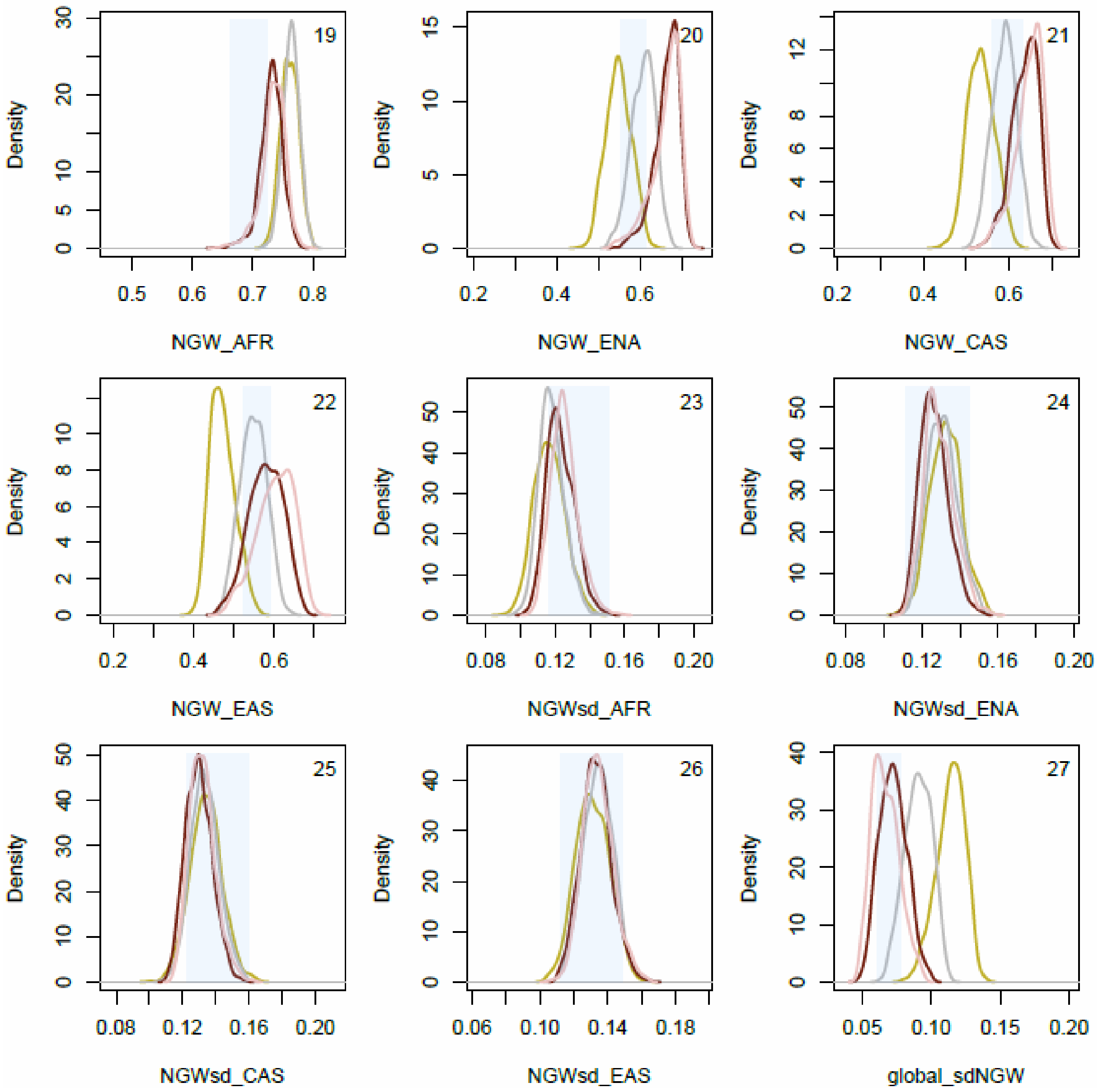

**Supplementary Figure 12 cont.**

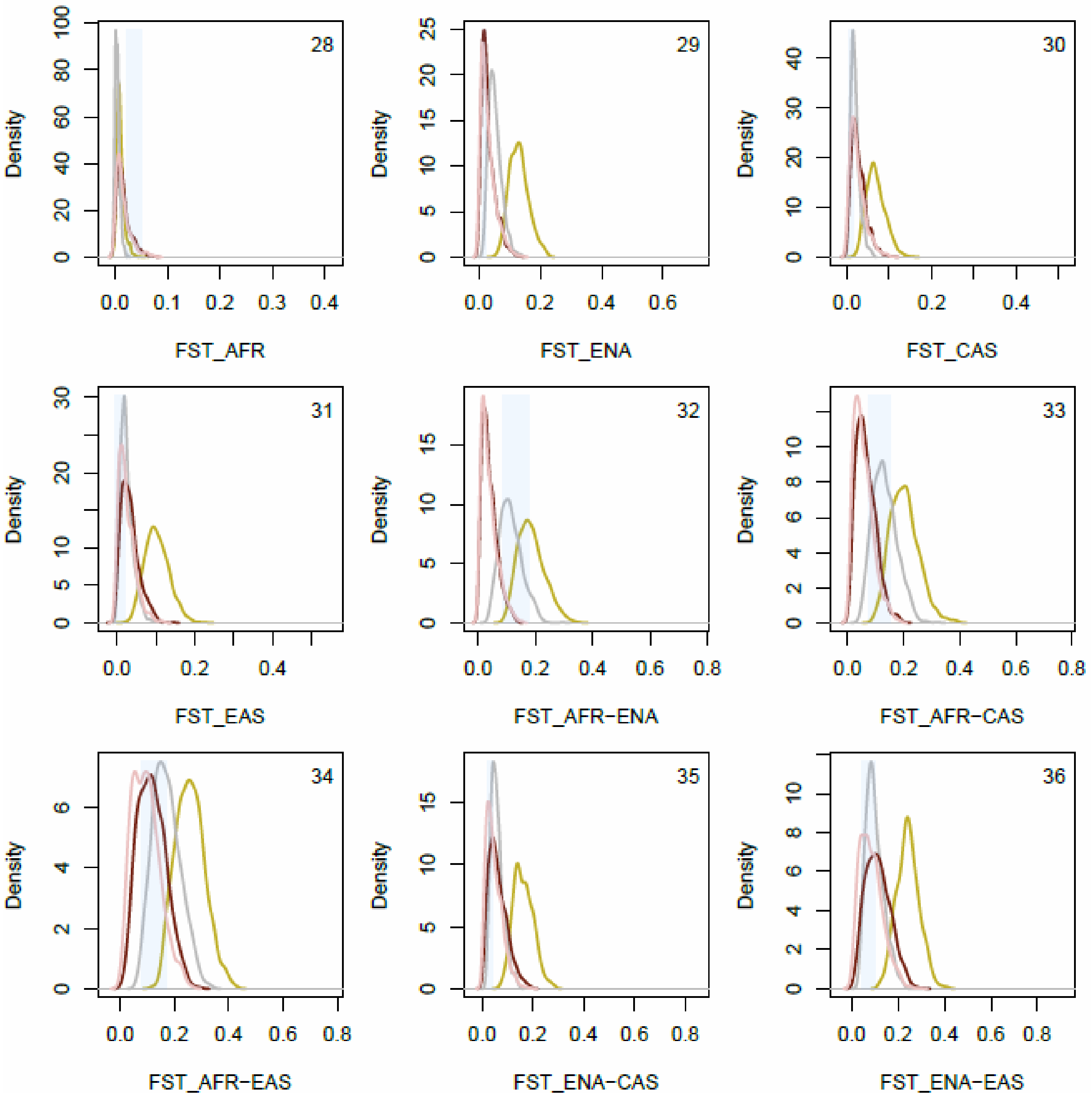

**Supplementary Figure 12 cont.**

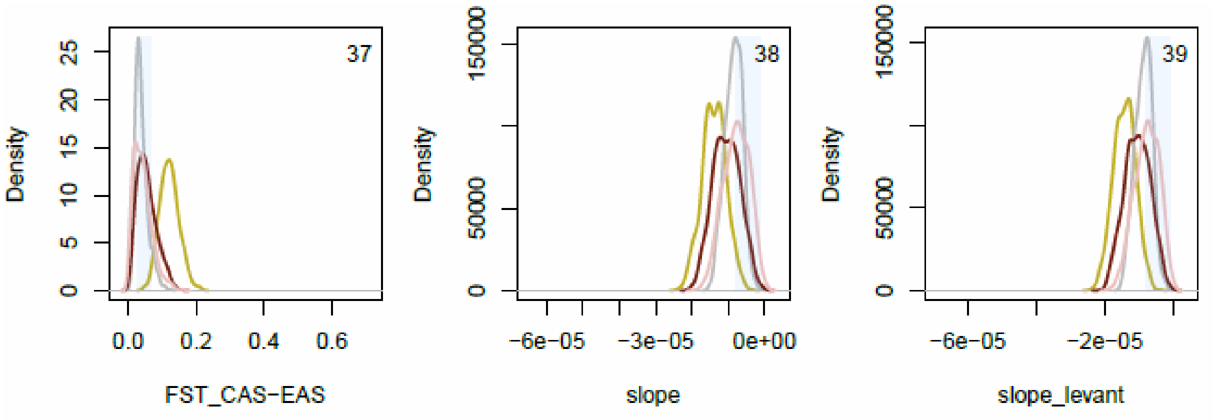


**Figure S12.** Distributions of summary statistics in the 10% best simulations under two models with LGM-induced range contraction and 150 km-wide demes, either with or without LDD, and the same models but with larger deme dimension, 250x250 km. Shaded area represents the distribution of values of each SS among the 1,000 resampled observed datasets. SS are fully described in Table S4. The x-axis corresponds to the prior range of the summary statistics.

## Supplementary Tables

**Table S1.** Power of model choice method to recover the right model using the multivariate logistic regression (Beaumont 2008) and two assignment thresholds.

| | *noLDDnoRC* | | *noLDDRC* | | *LDDnoRC* | | *LDDRC* | | *Not Assigned* | |
|---|---|---|---|---|---|---|---|---|---|---|
| *Threshold*[a] / *Model* | 0.50 | 0.85 | 0.50 | 0.85 | 0.50 | 0.85 | 0.50 | 0.85 | 0.50 | 0.85 |
| *noLDDnoRC* | 0.995 | 0.996 | 0.004 | 0.004 | 0 | 0 | 0.001 | | 0 | 0.002 |
| *noLDDRC* | 0.004 | 0.004 | 0.995 | 0.995 | 0 | 0 | 0.001 | 0.001 | 0 | 0 |
| *LDDnoRC* | 0 | 0 | 0 | 0 | 0.868 | 0.953 | 0.132 | 0.047 | 0 | 0.317 |
| *LDDRC* | 0 | 0 | 0.002 | 0.003 | 0.119 | 0.026 | 0.879 | 0.971 | 0 | 0.279 |

We performed model selection on 1,000 PODS generated under each model and reference tables encompassing 99,000 simulations per evolutionary scenario. Values in the table stand for the proportion of PODS assigned to the correct model when a model posterior probability exceeds either the values of 0.50 or 0.85, used as threshold for model assignment (supplementary Fig. S1). Whenever a PODS did not reach a model posterior probability larger than predefined threshold it was allocated to the "Not Assigned" category (see Material and Methods for more details).

**Table S2.** Power of model choice method to recover the right model using the multivariate logistic regression (Beaumont 2008) and two assignment thresholds.

| Model \ Threshold | *LDDnoRC* | | *LDDnoRCep* | | *LDDnoRCop* | | *LDDRC* | | *LDDRCep* | | *LDDRCop* | | Not assigned | |
|---|---|---|---|---|---|---|---|---|---|---|---|---|---|---|
| | 0.50 | 0.85 | 0.50 | 0.85 | 0.50 | 0.85 | 0.50 | 0.85 | 0.50 | 0.85 | 0.50 | 0.85 | 0.50 | 0.85 |
| *LDDnoRC* | 0.795 | 0.916 | 0.068 | 0.029 | 0.004 | 0 | 0.106 | 0.044 | 0.008 | 0.002 | 0.018 | 0.009 | 0.057 | 0.546 |
| *LDDnoRCep* | 0.085 | 0.036 | 0.835 | 0.934 | 0 | 0 | 0.009 | 0.006 | 0.066 | 0.022 | 0.005 | 0.002 | 0.06 | 0.365 |
| *LDDnoRCop* | 0.003 | 0.002 | 0 | 0 | 0.879 | 0.975 | 0 | 0 | 0 | 0 | 0.118 | 0.023 | 0.018 | 0.391 |
| *LDDRC* | 0.069 | 0.025 | 0.005 | 0.004 | 0.002 | | 0.822 | 0.931 | 0.074 | 0.029 | 0.027 | 0.012 | 0.06 | 0.479 |
| *LDDRCep* | 0.001 | 0 | 0.041 | 0.018 | 0 | 0 | 0.083 | 0.021 | 0.871 | 0.958 | 0.004 | 0.003 | 0.034 | 0.327 |
| *LDDRCop* | 0.005 | 0 | 0.001 | 0 | 0.149 | 0.038 | 0.010 | 0.002 | 0.002 | 0 | 0.832 | 0.960 | 0.021 | 0.394 |

We performed model selection on 1,000 PODS generated under each model and reference tables containing 19,000 simulations per scenario. Values in the table stand for the proportion of PODS assigned to the correct model when the model posterior probability required is either >0.5 or >0.85 (supplementary Fig. S5). Whenever one PODS did not reach a model posterior probability larger then predefined thresholds, it was allocated to the “Not Assigned” category (see Material and Methods for more details).

**Table S3.** Prior distributions of the historical and demographic parameters included in the four models tested in this study.

| Parameter | Distribution | *noLDDnoRC* | *noLDDRC* | *LDDnoRC* | *LDDRC* |
|---|---|---|---|---|---|
| Emergence of modern humans ($T_{STARTEXP}$) | Uniform [80,000-150,000] | X | X | X | X |
| Out of Africa ($T_{OOA}$) | Uniform [40,000-80,000] | X | X | X | X |
| Onset range contraction ($T_{SCONTR}$) | 25,000 | | X | | X |
| End range contraction ($T_{ECONTR}$) | 22,000 | | X | | X |
| Onset re-expansion ($T_{REXP}$) | 18,000 | | X | | X |
| Ancestral size ($Ne_{ANC}$) | Uniform [5000 – 25,000] | X | X | X | X |
| Growth rate (*r*) | Uniform [0.2 – 1.0] | X | X | X | X |
| Migration rate (*m*) | Uniform [0.05 – 0.3] | X | X | X | X |
| Carrying capacity (*K*) | Uniform [50 – 2500] | X | X | X | X |
| Carrying capacity during Neolithic (*KNeo*) | Uniform [2500-7500] | # | # | # | # |
| Time for the increase in population size driven by the Neolithic (*tNeo*) | 8,000 | # | # | # | # |
| LDD proportion ($LDD_{PROP}$) | Uniform [0.01 – 0.05] | | | X | X |
| Gamma shape parameter – LDD distance (α) | Uniform [0.5-2] | | | X | X |
| Average number of demes travelled by LDD migrants (μ) | Uniform [2-6] | | | X | X |
| LDD max distance travelled by LDD migrants | 6 demes | | | X | X |
| Origin of the expansion | Ethiopia (Addis Ababa) | X | X | X | X |
| Mutation rate ($STR_{MUTRATE}$) | Uniform [0.00005 – 0.0003] | X | X | X | X |

Times are shown in years, assuming a generation time of 25 years and population sizes ($Ne_{ANC}$, *K* and *KNeo*) are expressed in number of diploid individuals. # - parameters only present in the four models

in which the Neolithic period is mimicked by an increase in population size (see Material and Methods for more details).  X – presence/absence of a parameter in the different scenarios.

**Table S4.** Summary statistics (SS) used to perform model choice. To reduce the number of SS, measures of population genetic diversity and differentiation were averaged over populations belonging to four different groups: AFR- Africa, ENA – Europe and North Africa, CAS – central Asia and East Asia. Figure 1D shows how the 22 human populations were distributed into these groups.

| Summary statistic name | Description |
|---|---|
| K_AFR | Average number of alleles (K) in populations from the African group |
| K_ENA | Average K in populations from the Europe and North African group |
| K_CAS | Average K in populations from the Central Asian group |
| K_EAS | Average K in populations from the East Asian group |
| Ksd_AFR | Over loci K standard deviation (Ksd) averaged in populations from the African group |
| Ksd_ENA | Average Ksd in populations from the Europe and North African group |
| Ksd_CAS | Average Ksd in populations from the Central Asian group |
| Ksd_EAS | Average Ksd in populations from the East Asian group |
| global_sdK | Over loci standard deviation of K in all pooled populations |
| H_AFR | Average heterozygosity (H) in populations from the African group |
| H_ENA | Average H in populations from the Europe and North African group |
| H_CAS | Average H in populations from the Central Asian group |
| H_EAS | Average H in populations from the East Asian group |
| Hsd_AFR | Over loci H standard deviation (Hsd) averaged in populations from the African group |
| Hsd_ENA | Average Hsd in populations from the Europe and North African group |
| Hsd_CAS | Average Hsd in populations from the Central Asian group |
| Hsd_EAS | Average Hsd in populations from the East Asian group |

| | |
|---|---|
| global_sdH | Over loci standard deviation of H in all pooled populations |
| NGW_AFR | Average Garza-Williamson GW* statistic (NGW) in populations from the African group |
| NGW_ENA | Average NGW in populations from the Europe and North African group |
| NGW_CAS | Average NGW in populations from the Central Asian group |
| NGW_EAS | Average NGW in populations from the East Asian group |
| NGWsd_AFR | Over loci standard deviation of NGW (NGWsd) in populations from the African group |
| NGWsd_ENA | Average NGWsd in in populations from the Europe and North African group |
| NGWsd_CAS | Average NGWsd in populations from the Central Asian group |
| NGWsd_EAS | Average NGWsd in populations from the East Asian group |
| global_sdNGW | Standard deviation of the Garza-Williamson GW* statistic in all pooled populations |
| FST_AFR | Average pairwise FSTs between populations within the African group |
| FST_ENA | Average pairwise FSTs between populations from the European and North African group |
| FST_CAS | Average pairwise FSTs between populations within the Central Asian group |
| FST_EAS | Average pairwise FSTs between populations within the East Asian group |
| FST_AFR-ENA | Average pairwise FSTs between populations from the African and Europe and North African groups. |
| FST_AFR-CAS | Average pairwise FSTs between populations from the African and Central Asian groups |
| FST_AFR-EAS | Average pairwise FSTs between populations from the African and East Asian groups |
| FST_ENA-CAS | Average pairwise FSTs between populations from the Europe and North African and Central Asian groups |
| FST_ENA-EAS | Average pairwise FSTs between populations from the Europe and North African and East Asian groups |

| | |
|---|---|
| FST_CAS-EAS | Average pairwise FSTs between populations from the Central and East Asian groups |
| slope | Slope of the linear regression of heterozygosity against geographic distance from East Africa, assuming that migrants could cross water bodies up to 6 demes wide. |
| slope_levant | Slope of the linear regression of heterozygosity against geographic distance from East Africa, assuming that migrants could only spread over continuous landmasses. |

## Supplementary References

Beaumont M 2008. Joint determination of topology, divergence time, and immigration in population trees. In: Matsumura S, Renfrew PFC, editors. Simulation, genetics and human prehis- tory. Cambridge:McDonald Institute for Archeological Research: 134–154.

Daub JT, Dupanloup I, Robinson-Rechavi M, Excoffier L 2014. Inference of evolutionary forces acting on human biological pathways. BioRxiv:10.1101/009928.
